# Type 1 diabetes vaccine candidates promote human Foxp3^+^Treg induction in humanized mice

**DOI:** 10.1038/ncomms10991

**Published:** 2016-03-15

**Authors:** Isabelle Serr, Rainer W. Fürst, Peter Achenbach, Martin G. Scherm, Füsun Gökmen, Florian Haupt, Eva-Maria Sedlmeier, Annette Knopff, Leonard Shultz, Richard A. Willis, Anette-Gabriele Ziegler, Carolin Daniel

**Affiliations:** 1 Institute for Diabetes Research, Independent Young Investigator Group Immune Tolerance in Type 1 Diabetes, Helmholtz Diabetes Center at Helmholtz Zentrum München, Heidemannstrasse 1, 80939 München, Germany; 2 Deutsches Zentrum für Diabetesforschung (DZD), Ingolstädter Landstrasse 1, 85764 München, Germany; 3 Institute for Diabetes Research, Helmholtz Diabetes Center at Helmholtz Zentrum München, Klinikum rechts der Isar, Technische Universität München, Heidemannstrasse 1, 80939 München, Germany; 4 The Jackson Laboratory, 600 Main Street, Bar Harbor, Maine 04609, USA; 5 Emory Vaccine Center, NIH Tetramer Core Facility, 201 Dowman Drive, Atlanta, Georgia 30322, USA

## Abstract

Immune tolerance is executed partly by Foxp3^+^regulatory T (Treg) cells, which suppress autoreactive T cells. In autoimmune type 1 diabetes (T1D) impaired tolerance promotes destruction of insulin-producing β-cells. The development of autoantigen-specific vaccination strategies for Foxp3^+^Treg-induction and prevention of islet autoimmunity in patients is still in its infancy. Here, using human haematopoietic stem cell-engrafted NSG-HLA-DQ8 transgenic mice, we provide direct evidence for human autoantigen-specific Foxp3^+^Treg-induction *in vivo*. We identify HLA-DQ8-restricted insulin-specific CD4^+^T cells and demonstrate efficient human insulin-specific Foxp3^+^Treg-induction upon subimmunogenic vaccination with strong agonistic insulin mimetopes *in vivo*. Induced human Tregs are stable, show increased expression of Treg signature genes such as *Foxp3, CTLA4, IL-2Rα* and *TIGIT* and can efficiently suppress effector T cells. Such Foxp3^+^Treg-induction does not trigger any effector T cells. These T1D vaccine candidates could therefore represent an expedient improvement in the challenge to induce human Foxp3^+^Tregs and to develop novel precision medicines for prevention of islet autoimmunity in children at risk of T1D.

## Abstract (web summary)

Type 1 diabetes is associated with the loss of self-tolerance to the insulin-producing β-cells in the pancreas. Here the authors show that vaccination with insulin mimetopes can induce human insulin-specific regulatory T cells to mediate tolerance in a humanized mouse model.

## Graphical abstract

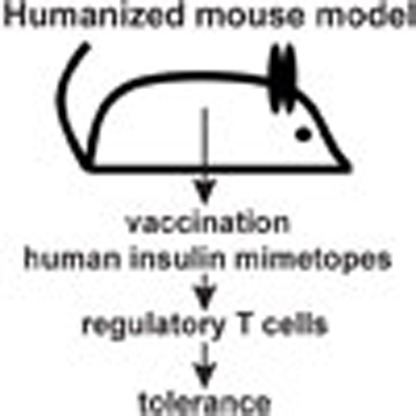

Type 1 diabetes (T1D) afflicts millions of people worldwide and is a severe chronic autoimmune disease characterized by the progressive loss of self-tolerance to insulin-producing pancreatic β-cells^1^. The incidence of T1D is rising dramatically especially in young children^2^. T1D and other autoimmune diseases are thought to develop when T cells with specificity for weakly binding T-cell receptor (TCR) agonists, which may include self-antigens, evade thymic negative selection and then mount a peripheral autoimmune attack^3^,^4^,^5^,^6^,^7^. In children, the appearance of multiple islet autoantibodies indicates the onset of islet autoimmunity (pre-T1D)^8^. Insulin autoantibodies are often the first to appear thereby highlighting the contribution of insulin in initiating T1D autoimmunity^9^.

Regulatory T (Treg) cells are pivotal in preventing autoimmunity. Impairments in Treg numbers, function and induction critically contribute to autoimmune destruction in T1D. Tregs are characterized by the expression of the high-affinity interleukin-2 (IL-2) receptor α-chain (*IL-2Rα*) and the X-linked gene forkhead box P3 (*Foxp3*), encoding the transcription factor Foxp3, which acts as a lineage specification factor for the development and function of CD4^+^CD25^+^Tregs^10^,^11^,^12^,^13^. The essential function of human Foxp3^+^Tregs to avoid autoimmunity is illustrated by the fatal autoimmune disease IPEX (immunodysregulation, polyendocrinopathy, enteropathy and X-linked syndrome), which is caused by mutations in the *Foxp3* gene.

Foxp3^+^Tregs have attracted attention as they can ‘tame' their autoreactive counterparts by direct contact-dependent inhibition of antigen-presenting cells (APCs) and effector T cells or by releasing inhibitory cytokines such as TGFβ or IL-10. Tregs maintain their regulatory functions for a long period of time even in the absence of antigens that induced their generation and are stable and transferable^14^, thereby permitting the prospective induction of these cells to prevent unwanted immunity. We are focusing on novel strategies using optimized variants of critical autoantigens for Foxp3^+^Treg induction since Tregs bear the promise of specifically targeting the harmful effects of peripheral autoreactive T cells to control autoimmunity such as that observed in T1D while preserving the ability of the immune system to fight off infections^15^,^16^,^17^,^18^. Optimal *in vivo* induction of stable murine Foxp3^+^Tregs requires the subimmunogenic delivery of strongly agonistic TCR ligands to naive CD4^+^T cells^16^,^17^,^19^,^20^,^21^. By contrast, even high immunogenic doses of weakly agonistic ligands fail to induce stable Foxp3^+^Tregs^17^,^22^. The most efficient Foxp3^+^Treg induction is achieved in T cells that proliferated least extensively^19^. Specific Foxp3^+^Treg induction in the context of autoimmunity could allow modulating the immune response for clinical benefit while limiting long-term immune suppression.

T1D mouse models as non-obese diabetic (NOD) mice showed that insulin functions as an essential autoantigen^23^,^24^. In humans and mice, T cell responses to insulin are highly focused on a human leukocyte antigen (HLA)-DQ8- or murine IA^g7^-restricted segment of the insulin-B-chain comprising residues 9–23 and the human epitope is identical to that of mouse insulin^25^,^26^,^27^. Initial murine studies using subimmunogenic delivery of natural insulin B-chain epitopes show only a limited Treg induction efficacy and a slight delay in T1D progression^17^. As one possible means to explain the poor efficacy of Treg induction by natural insulin B-chain epitopes in murine T1D, it has been indicated that the insulin-B-chain peptide is presented by I-A^g7^ in a low-affinity binding register, which results in weak-agonistic activity of the peptide presented by the major histocompatibility complex (MHC)II (refs 7, 28). To efficiently induce insulin-specific Foxp3^+^Tregs that could interfere with the development of T1D in NOD mice, we devised a strongly agonistic mimetope of the natural insulin-B-chain-epitope (21E-22E) with improved MHCII-binding^7^ and showed that its sub-immunogenic delivery promoted efficient Foxp3^+^Treg induction and T1D protection for 40 weeks and longer^17^. Importantly, crystal structures of the human T1D susceptibility HLA-DQ8 allele and the homologous molecule in NOD mice, I-A^g7^, reveal striking structural overlap between the MHC-peptide binding pockets^29^, which suggests similar peptide presentation events of insulin-epitopes in human T1D. Accordingly, a recent study provides evidence that insulin B:9-23-reactive CD4^+^T cells are present in the peripheral blood of T1D patients and that the immunogenic register of this peptide has low-affinity binding to HLA-DQ8 (ref. 30). Moreover, T1D risk may be related to how an *HLA-DQ* genotype determines the balance of T-cell inflammatory versus regulatory responses to insulin, having implications for insulin-specific therapies to prevent T1D (ref. 31).

Currently, the majority of strategies approved by the FDA for autoimmune diseases have focused on non-antigen-specific immune suppression. Although this was found to be partially effective in inhibiting autoreactivity, these compounds have numerous side effects and long-term treatment remains challenging. Strategies that promote autoantigen-specific Treg induction will permit the specific blockade of the deleterious effects of autoimmune destruction while maintaining the ability of the immune system to clear non-autoantigens. While promising results have been obtained in mice, in man the development of autoantigen-specific Foxp3^+^Treg induction strategies is still in its infancy. It is currently unclear whether concepts established for efficient murine *in vivo* Foxp3^+^Treg induction will be translatable to the human immune system, especially in the context of autoimmune diseases such as T1D. Further studies are needed that provide mechanistic insights for the *in vivo* induction of human autoantigen-specific Foxp3^+^Tregs. As an excellent accessible system permitting predictive *in vivo* immunology research, here we used human haematopoietic stem cell (HSC)-engrafted NOD-Scid-IL2-receptor-γ-chain knockout (NSG)-HLA-DQ8 transgenic mice and newly established autoantigen-specific Treg induction.

We provide first direct evidence that a set of two novel human insulin mimetopes promotes human Foxp3^+^Treg induction in human-HSC-engrafted NSG-HLA-DQ8 transgenic mice *in vivo*. Such induced Tregs from humanized mice are stable over prolonged periods of time, present with robust suppressive capacities and harbour high abundance of Treg signature genes such as *Foxp3, CTLA4, IL-2Rα* and *TIGIT* in the absence of effector T-cell responses. These T1D vaccine candidates could critically contribute to the development of efficient autoantigen-specific Treg induction strategies for prevention of islet autoimmunity in children at risk of developing T1D.

## Results

### Agonistic activity of insulin mimetopes in CD4^+^T cells

To define optimal conditions for human insulin-specific Foxp3^+^Treg induction we tested agonistic activities of four insulin-B-chain-10-23 mimetopes. Peptide selections were made first based on the finding that insulin-B:10-23 peptide variants with a mutation of arginine (R) to glutamic acid (E) at position 22 and/or including a change to glycine (G) at position 21 (ins.mim.2=21G-22E; ins.mim.3=21E-22E) were more potent in stimulating murine insulin-specific CD4^+^T cells^7^,^17^,^28^ and stimulated human insulin-specific CD4^+^T cells^30^,^31^. The use of either E or G at position 21 was included based on findings in NOD mice that insulin B:9–23-specific CD4^+^T cells can be divided into types A and B T cells. Both recognize the peptide bound to IA^g7^ in register 3. However, type A T cells prefer the glutamic acid at position 21 as TCR-binding residue while type B cells prefer glycine 21 (ref. 28).

Second, we set up two novel human insulin mimetopes with mutations at position 22 to glutamic acid (E) together with position 21 being E or G and an additional mutation of position 14 from alanine (A) to glutamic acid (E) (ins.mim.1=14E-21G-22E; ins.mim.4=14E-21E-22E). The mutation at position 14 was included since structural analyses of a human insulin-peptide-HLA-DQ8 complex had suggested that glutamic acid (=E) is preferred over alanine at the first MHC-anchor^29^ (Supplementary Fig. 1 for peptide sequences).

**Figure 1:**
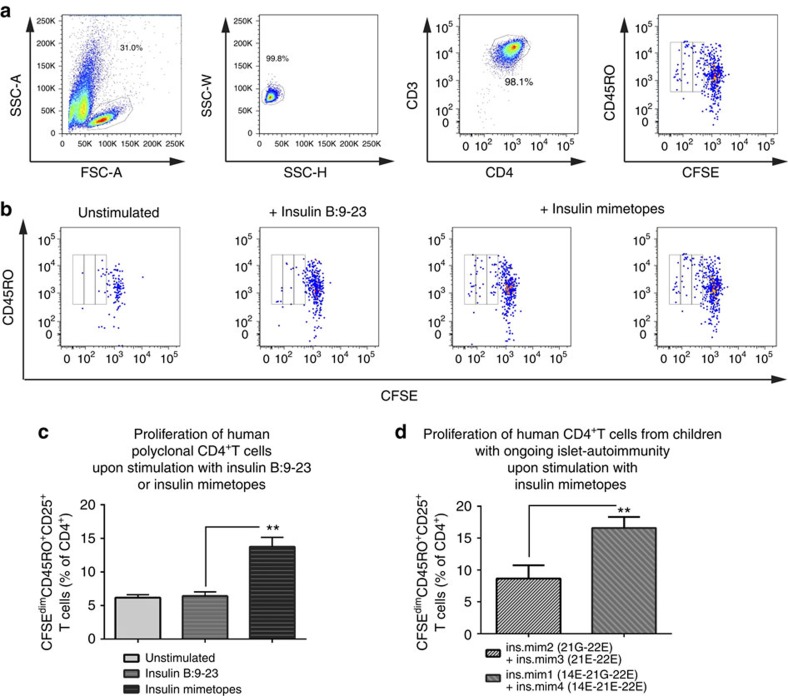
Agonistic activity of insulin mimetopes in human polyclonal CD4^+^T cells required for Foxp3^+^Treg induction. (**a**) Representative FACS plots for the identification of proliferating human polyclonal CD4^+^CD3^+^T cells by CFSE-dilution in CD45RO^+^CD4^+^CD25^+^T cells (left to right). (**b**) Representative FACS plots of CFSE-dilution profiles from human polyclonal CD4^+^T cells purified from children with or without pre-T1D either left untreated, stimulated with the natural insulin B:9-23 epitope (1 μg ml^−1^) or a set of four insulin-B-chain-10-23-mimetopes (shown are two staining examples of cells stimulated with ins.mim.2=21G-22E; ins.mim.3=21E-22E; ins.mim.1=14E-21G-22E; ins.mim.4=14E-21E-22E, final concentration at 1 μg ml^−1^). Proliferating CFSE^dim^CD4^+^CD25^+^CD45RO^+^T cells were considered as responding T cells. (**c**) Percentages of divided human CFSE^dim^CD4^+^CD25^+^CD45RO^+^T cells. Bars represent the means±s.e.m. (*n*=8) from duplicate wells of eight children performed in four independent experiments. **P<0.01 (Student's *t*-test). (**d**) Percentages of divided human CFSE^dim^CD4^+^CD45RO^+^T cells upon stimulation with a combination of ins.mim.2=21G-22E; ins.mim.3=21E-22E or a combination of ins.mim.1=14E-21G-22E; ins.mim.4=14E-21E-22E. Bars represent the means±s.e.m. (*n*=6) from duplicate wells of six children done in three independent experiments. **P<0.01 (Student's *t*-test).

Proliferative responses were assessed using polyclonal CFSE-labelled CD4^+^T cells from eight islet autoantibody positive *HLA-DQ8*^+^ children. Comparisons were made upon stimulation with either the natural insulin-B-chain-epitope or a set of insulin-B-chain mimetopes or as controls left untreated. When the proportion of cells with diluted CFSE-label was determined, the insulin mimetopes showed increased stimulatory capacities when compared with the natural insulin-B-chain epitope (unstimulated: 6.2±0.2 versus insulin B:9-23: 6.4±0.3 versus insulin mimetopes: 13.7±1.4 CFSE^dim^CD45RO^+^CD25^+^T cells in % of CD4^+^T cells, *P*<0.01, Fig. 1).

Moreover, a combination of ins.mim.1=14E-21G-22E and ins.mim.4=14E-21E-22E resulted in significantly enhanced stimulation when compared with ins.mim.2=21G-22E and ins.mim.3=21E-22E) either in CD4^+^T cells from non-diabetic children with ongoing islet autoimmunity (Fig. 1d, *P*<0.01) or without autoimmunity (Supplementary Fig. 2).

### *Ex vivo* identification of human insulin-specific Tregs

**Figure 2:**
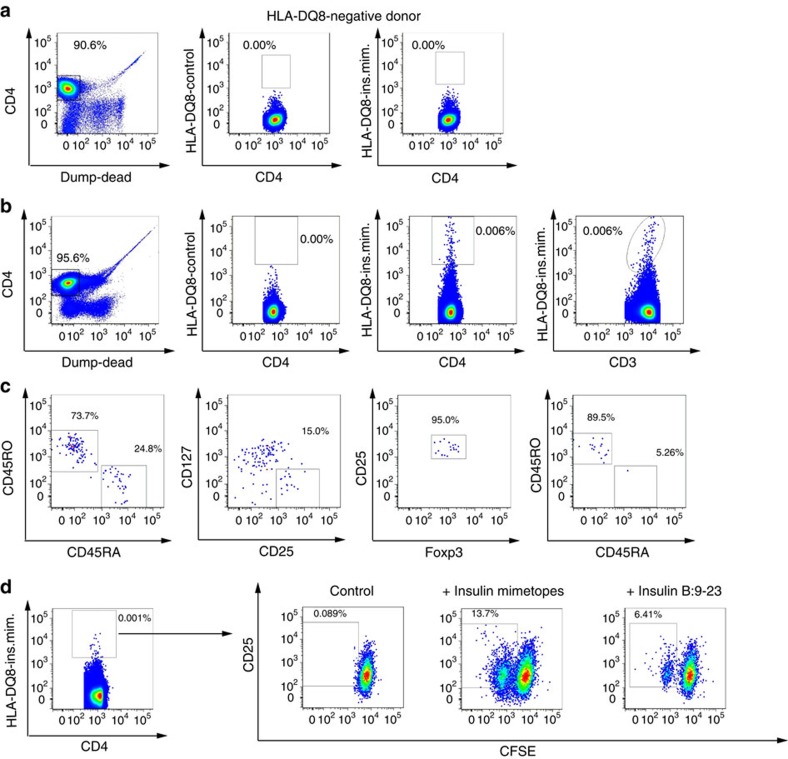
*Ex vivo* identification of human HLA-DQ8-restricted insulin-specific regulatory T cells. Human CD4^+^T cells were analysed by FACS, first gating on live, CD14^−^, CD19^−^, CD8a^−^, CD11b^−^, CD4^+^ (left; **a**,**b**) and CD3^+^, followed by examination of tetramer binding. (**a**) Representative FACS plots for the direct *ex vivo* identification of HLA-DQ8-restricted insulin-specific CD4^+^T cells. Control staining was performed to assess the quality and specificity of the tetramer staining using a combination of two control tetramers fused to irrelevant peptides (centre) or using CD4^+^T cells from an *HLA-DQ8*-negative individual (right). (**b**) Representative FACS plots for the quality of HLA-DQ 8-restricted insulin-specific tetramer staining gating against CD4 (centre) and CD3 (right). (**c**) Representative FACS plots for the phenotypic characterization of HLA-DQ8-restricted insulin-specific CD4^+^T cells based on CD45RA versus CD45RO expression (memory status) and of insulin-specific Foxp3^+^Tregs based on CD127^low^CD25^high^ and Foxp3^high^ expression. (**d**) Re-stimulation of sorted and purified tetramer^+^CD4^+^T cells (example plot on the left) either left untreated (=control, left) or stimulated with insulin mimetopes (ins.mim.1,2,3,4 at final 10 ng ml^−1^, middle plot), or with insulin B:9-23 (100 ng ml^−1^, right), as assessed by analysing the dilution of the CFSE label and CD25 expression.

Next, based on their enhanced stimulatory potential, agonistic activity and in accordance with identified crystal structures^29^ we employed 14E-21G-22E (ins.mim.1) and 14E-21E-22E (ins.mim.4) for the development of insulin-specific HLA-DQ8-tetramers. CD4^+^T-cell enrichment before flow cytometric enumeration distinctly increased sensitivity of detection of insulin-specific CD4^+^T cells. Virtually no tetramer^+^CD4^+^T cells were detected with the HLA-DQ8 control tetramers and by using CD4^+^T cells from an *HLA-DQ8*-negative donor (Fig. 2a,b). By contrast, insulin mimetope-specific CD4^+^T cells were readily identified *ex vivo* using HLA-DQ8 insulin mimetope-specific tetramers, and frequencies of tetramer^+^CD4^+^T cells were correlated with CD3 expression (second right and right plots in Fig. 2b).

To permit for the first time the direct *ex vivo* identification of human HLA-DQ8-restricted insulin-specific Foxp3^+^Tregs we used pre-enriched CD4^+^T cells and newly developed settings to combine tetramer stainings with multiparameter flow cytometry and intracellular Foxp3 staining (Fig. 2c).

To verify the specificity of the tetramer^+^CD4^+^T cells for the insulin mimetope and insulin B:9–23 itself, tetramer^+^cells were sort-purified and expanded in a polyclonal fashion. Re-stimulation of CD4^+^T cells with the insulin mimetopes induced rapid proliferation, as determined by dilution of the CFSE label (Fig. 2d). The CD4^+^T cells likewise responded to the natural insulin B:9–23 epitope, albeit to a lesser extent (right plot in Fig. 2d).

### Insulin-specific Foxp3^+^Tregs and autoimmune progression

**Figure 3:**
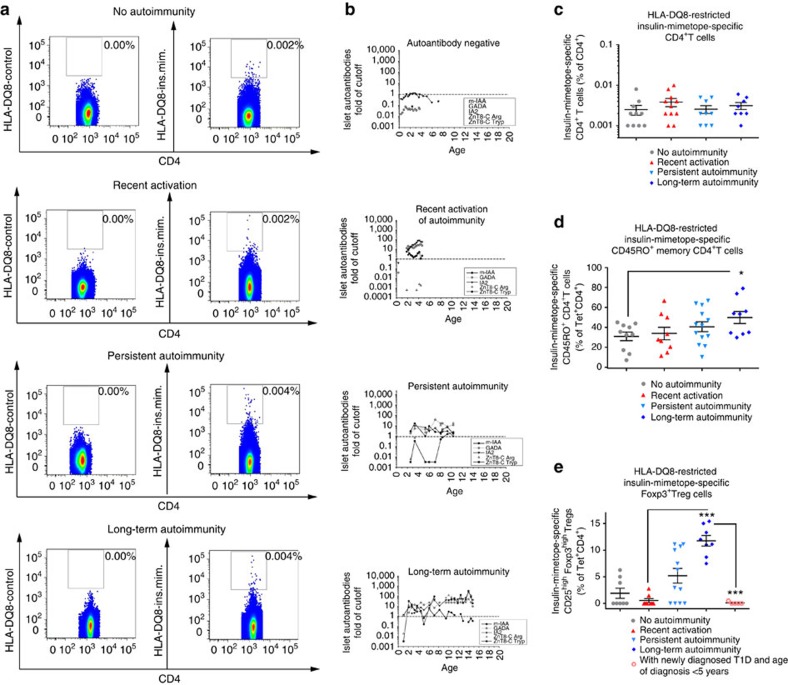
Frequency of insulin mimetope-specific Foxp3^+^Tregs in children with recent onset of islet autoimmunity. (**a**) Representative set of FACS plots for the identification of HLA-DQ8-restricted insulin mimetope-specific CD4^+^T cells with control (left) and insulin mimetope-specific tetramer (right) staining using CD4^+^T cells purified from *HLA-DQ8*^*+*^ children without autoimmunity (islet autoantibody negative), with recent onset of autoimmunity (recent activation=multiple autoantibodies for ≤5 years), persistent autoimmunity (multiple autoantibodies for >5 to ≤10 years) and longterm autoimmunity (multiple autoantibodies >10 years without T1D). (**b**) Representative autoantibody profiles shown as the fold cutoff value for each disease stage. (**c**) Frequency of HLA-DQ8-restricted insulin-specific tetramer^+^CD4^+^T cells in children without autoimmunity (islet autoantibody negative, *n*=10), with recent onset of autoimmunity (recent activation=multiple autoantibodies for ≤5 years, *n*=9), persistent autoimmunity (multiple autoantibodies for >5 to ≤10 years, *n*=13), and long-term autoimmunity (multiple autoantibodies >10 years without T1D, *n*=10). (**d**) Frequency of HLA-DQ8-restricted insulin-specific memory tetramer^+^CD45RO^+^CD4^+^T cells in children without autoimmunity (islet autoantibody negative, *n*=10), with recent onset of autoimmunity (recent activation=multiple autoantibodies for ≤5 years, *n*=9), persistent autoimmunity (multiple autoantibodies for >5 to ≤10 years, *n=*13), and longterm autoimmunity (multiple autoantibodies >10 years without T1D, *n*=10) (**e**) Frequency of HLA-DQ8-restricted insulin-specific tetramer^+^CD127^low^CD25^high^ CD4^+^Foxp3^high^ Tregs in children without autoimmunity (islet autoantibody negative, *n*=8), with recent onset of autoimmunity (multiple autoantibodies for ≤5 years, *n*=8), persistent autoimmunity (multiple autoantibodies for >5 to ≤10 years, *n*=12), longterm autoimmunity (multiple autoantibodies for >10 years, *n*=5), or newly diagnosed type 1 diabetes with very early disease manifestation (age at diagnosis ≤5 years, *n*=5). Data are presented as the mean±s.e.m. from 10 independent experiments. *P<0.05 and ***P<0.001 (Student's *t*-test).

HLA-DQ8-restricted insulin mimetope-specific CD4^+^T cells were identified in children without and with various durations of islet autoimmunity (disease categories: no autoimmunity: islet autoantibody negative, recent activation: multiple islet autoantibodies for <5 years and long-term autoimmunity: multiple islet autoantibodies >10 years, Fig. 3a,c). Likewise such T cells were found in children with recent-onset or longterm T1D (Supplementary Fig. 3). At least 8 × 10^6^ and up to 40 × 10^6^ cells were acquired and HLA-DQ8-restricted insulin mimetope-specific CD4^+^T cells were detected in a range of 0.001–0.01% of CD4^+^T cells (see summary graph in Fig. 3c). These frequencies of insulin mimetope-specific CD4^+^T cells are in accordance with the range that has been estimated for islet-antigen-specific CD4^+^T cells, for example for proinsulin_76-90_-specific CD4^+^T cells the frequency has been estimated to be ∼1 in 300,000 PBMCs^32^,^33^. Phenotyping of HLA-DQ8-restricted insulin mimetope-specific CD4^+^T cells revealed an increase in the frequency of cells with a memory phenotype according to the duration of islet autoimmunity (Fig. 3d).

In young at-risk children, T1D can develop within several months of the appearance of autoantibodies. However, it may take more than a decade in some children^8^,^9^, supporting the concept of episodes of successful immune tolerance. Of interest, frequencies of insulin mimetope-specific Foxp3^+^Tregs were significantly lower in children with recent onset of autoimmunity than in children without autoimmunity (no autoimmunity versus recent onset of autoimmunity: 1.9±0.9 versus 0.5±0.4% of Tet^+^CD4^+^T cells, *P*<0.05, Fig. 3e). We identified enhanced frequencies of insulin mimetope-specific Foxp3^+^Tregs in non-diabetic children with longterm autoimmunity (long-term autoimmunity versus recent onset of autoimmunity: 11.7±0.9 versus 0.5±0.4 Foxp3^+^Tregs as a % of Tet^+^CD4^+^T cells, *P*<0.001, Fig. 3e), indicative of at least periods of successful ongoing immune regulation in such children. To further support a critical role of insulin mimetope-specific Tregs in delaying progression of islet autoimmunity to clinically overt T1D, frequencies of insulin mimetope-specific Tregs were found to be severely reduced in children with newly diagnosed T1D and a very early disease manifestation (age at diagnosis ≤5 years) (Fig. 3e). These findings underscore the rationale of inducing autoantigen-specific Tregs for delaying T1D progression.

### Agonistic activity of insulin mimetopes in CD4^+^T-cell clones

To determine agonistic activities of the individual insulin mimetopes we generated HLA-DQ8-restricted insulin mimetope-specific CD4^+^T-cell clones from children without islet autoimmunity or with various durations of islet autoimmunity. For the stimulation of human CD4^+^T cells and T-cell cloning we used HLA-DQ8 insulin mimetope-specific artificial APCs expressing insulinB:chain-10-23-mimetopes (14E-21G-22E (ins.mim.1) and 14E-21E-22E (ins.mim.4)) which were established using antibody-coupling beads, DQ-antibodies^34^ and unlabelled insulin mimetope-specific HLA-DQ8-tetramers. CD4^+^T cells responding to stimulation with insulin mimetope-specific artificial APCs were single-cell-sorted as CFSE^dim^CD25^high^CD4^+^T cells. In control experiments using HLA-DQ8-expressing artificial APCs fused to irrelevant peptides no dilution of the CFSE-label was observed (Supplementary Fig. 4).

**Figure 4:**
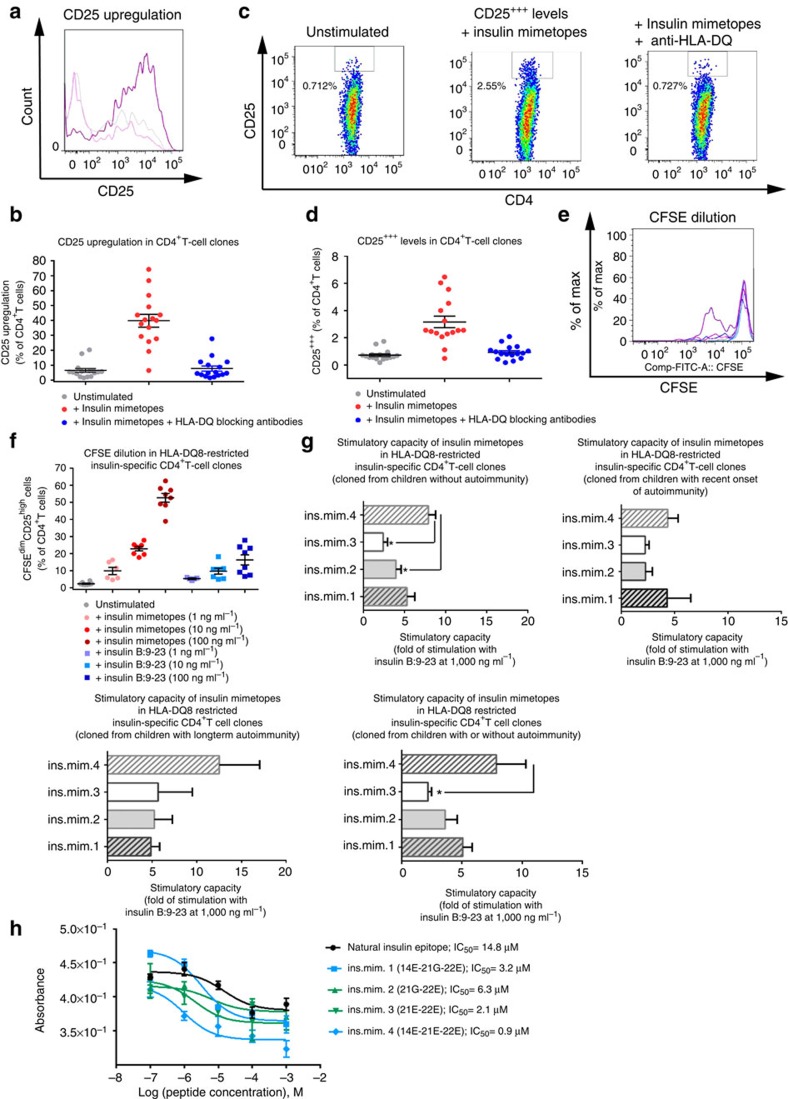
Agonistic activity of insulin mimetopes in human insulin-specific CD4^+^T-cell clones. (**a**) CD25 upregulation in CD4^+^T-cell clones left untreated or upon stimulation with insulin mimetopes (ins.mim.1,2,3,4 at 100 ng ml^−1^) with or without DQ-blocking antibodies (grey line: unstimulated control; dark pink line:+insulin mimetopes; light pink line:+insulin mimetopes+HLA-DQ blocking antibodies). (**b**) Summary graph for **a**. 16 CD4^+^T-cell clones: four from three subjects without ongoing autoimmunity; five from four subjects with recent onset of autoimmunity, seven from five individuals with long-term autoimmunity. (**c**) CD25^+++^ levels of an insulin-specific CD4^+^T-cell clone left unstimulated (left plot), stimulated with insulin mimetopes (middle plot) or with insulin mimetopes+HLA-DQ blocking antibodies (right plot). (**d**) Summary graph for **c**. Numbers of clones tested as in **b**. (**e**) CFSE-dilution profiles from insulin-specific CD4^+^T-cell clones (grey line: unstimulated; light blue:+insulin B:9-23 epitope (1 ng ml^−1^); blue line:+insulin B:9-23 epitope (10 ng ml^−1^); dark blue line:+insulin B:9-23 epitope (100 ng ml^−1^); light pink line:+insulin mimetopes (1 ng ml^−1^); pink line:+insulin mimetopes (10 ng ml^−1^); dark pink line:+insulin mimetopes (100 ng ml^−1^)). (**f**) Summary graph for **e**. Eight CD4^+^T-cell clones have been tested: two from two subjects without ongoing islet autoimmunity, two from two individuals with recent onset of autoimmunity, four from three individuals with long-term autoimmunity. (**g**) Summary graphs of stimulatory capacities of ins.mim.2=21G-22E; ins.mim.3=21E-22E; ins.mim.1=14E-21G-22E; ins.mim.4=14E-21E-22E (100 ng ml^−1^) in T-cell clones shown in fold of the stimulatory potential of the insulin B:9-23 (1,000 ng ml^−1^). Numbers of clones tested as in **b**. (**b**,**d**,**f**,**g**) Data represent the means±s.e.m. 16 clones (**b**,**d**,**g**) or eight clones (**f**) have been tested. *P<0.05 (Student's *t*-test). (**h**) *In vitro* binding of insulin B:9-23, ins.mim.1 (14E-21G-22E), ins.mim.2 (21G-22E), ins.mim.3 (21E-22E) and ins.mim.4 (14E-21E-22E) to HLA-DQ8. Competitive binding assays were carried out using a FITC-labelled reference GAD65 peptide and increasing concentrations of unlabelled insulin epitopes and mimetopes.

Insulin-specificity in growing CD4^+^T-cell clones was confirmed upon stimulation with insulin mimetopes in the presence or absence of DQ-blocking antibodies, analysed flow-cytometrically by CD25 upregulation (Fig. 4a,b) and confirmed by analyses of the highest CD25 levels (CD25^+++^ levels, Fig. 4c,d and Supplementary Fig. 5). All tested CD4^+^T-cell clones also responded to the natural insulin B:9-23 epitope albeit to a lower extent (Fig. 4e,f and Supplementary Fig. 5). These data show that T cells cloned from CD4^+^T cells responding to insulin-B-chain-10-23 mimetopes are likewise specific for the natural insulin B:9-23 epitope (Fig. 4e,f and Supplementary Fig. 5).

The set of four insulin-B-chain-10-23 mimetopes was used to assess their individual proliferative capacities (ins.mim.2=21G-22E; ins.mim.3=21E-22E; ins.mim.1=14E-21G-22E; ins.mim.4=14E-21E-22E) in generated insulin-specific CD4^+^T-cell clones (Fig. 4g). The stimulatory potential of the individual insulin mimetopes is shown in fold of the stimulation achieved with the natural insulin B:9-23 epitope. Irrespective of the presence or duration of autoimmunity (Fig. 4g) all insulin-variants were superior in stimulating insulin-specific CD4^+^T-cell clones. In particular ins.mim.4 (ins.mim.4=14E-21E-22E) presented with a significantly enhanced stimulatory capacity (*P*<0.05) when compared with ins.mim.2 (ins.mim.2=21G-22E) and ins.mim.3 (ins.mim.3=21E-22E). A summary of the stimulatory capacities of all tested insulin-specific CD4^+^T-cell clones is outlined in Fig. 4g. These findings are in accordance with our observations obtained from competitive *in vitro* HLA-DQ8 binding assays (Fig. 4h) where ins.mim.4 presented with the highest affinity to HLA-DQ8 (IC_50_=0.9 μM) when compared with ins.mim.3 (IC_50_=2.1 μM), ins.mim.2 (IC_50_=6.3 μM), ins.mim.1 (IC_50_=3.2 μM) and the natural insulin B:9-23 epitope (IC_50_=14.8 μM).

### Human insulin-specific Foxp3^+^Treg induction *in vitro*

**Figure 5:**
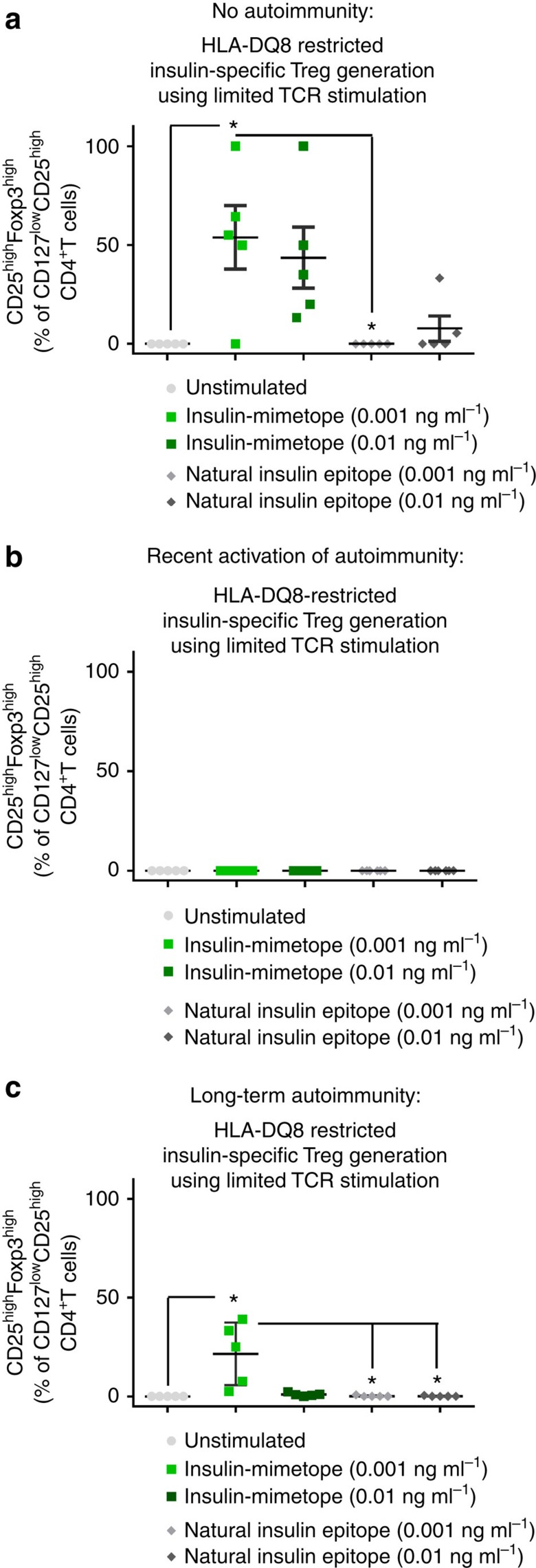
Human insulin-specific Foxp3^+^Treg induction using insulin mimetopes and subimmunogenic TCR stimulation. Comparison of human Treg induction potential of insulin mimetopes (ins.mim.1=14E-21G-22E; ins.mim.4=14E-21E-22E) and the natural insulin-B-chain 9-23 epitope using limited TCR stimulation in the presence of autologous CD304^+^ plasmacytoid, CD1c^+^ and CD141^+^ myeloid dendritic cells *in vitro* and human naive CD4^+^T cells purified from children with or without ongoing islet autoimmunity (no autoimmunity, *n*=5 per group (**a**); recent activation of autoimmunity, *n*=6 per group (**b**); longterm autoimmunity, *n*=5 per group (**c**)) from duplicate wells done in five independent experiments. Tregs were identified as CD4^+^CD3^+^CD127^low^CD25^+^T cells and then verified by intracellular staining for Foxp3. *P<0.05 (Student's *t*-test).

In agreement with their enhanced stimulatory potential, agonistic activity, in reference to identified categories of types A and B T cells^28^ and in accordance with identified crystal structures^29^ we used ins.mim.1 (14E-21G-22E) and ins.mim.4 (14E-21E-22E) to determine human insulin-specific Foxp3^+^Treg induction. We set up human *in vitro* Foxp3^+^Treg induction mimicking subimmunogenic TCR stimulation^16^,^35^. We developed a protocol for human insulin-specific Foxp3^+^Treg induction without TGFβ using premature withdrawal of TCR stimulation building up on murine studies^35^. Highly pure human naive CD4^+^T cells isolated from children with or without islet autoimmunity (Fig. 5a–c; disease categories: no autoimmunity, recent activation of autoimmunity and longterm autoimmunity) were used as a starting population (Supplementary Fig. 6 for gating example of human naive CD4^+^T cells). For antigen presentation during Treg induction autologous dendritic cells (DCs) were purified from PBMCs by depletion of CD14^+^ and CD19^+^ cells and subsequent positive selection of CD304^+^ plasmacytoid DCs and CD1c^+^ and CD141^+^ myeloid DCs.

We compared the *in vitro* Treg induction activity of the natural insulin-B-chain epitope with ins.mim.1 (14E-21G-22E) and ins.mim.4 (14E-21E-22E) (Fig. 5a–c). The combination of ins.mim.1=14E-21G-22E and ins.mim.4=14E-21E-22E showed best Treg induction as assessed by analysis of induced CD127^low^CD25^high^Foxp3^high^Tregs. Optimal Treg induction activity was seen in children without ongoing autoimmunity (insulin mimetope (ins.mim.1+4 final at 0.001 ng ml^−1^): 53.9±16.0 versus insulin-B-chain-epitope (0.001 ng ml^−1^): 0.0; *P*<0.05 or (0.01 ng ml^−1^): 7.7±6.4% of CD127^low^CD25^high^CD4^+^T cells, Fig. 5a).

### Stability of human Foxp3^+^Tregs induced *in vitro*

**Figure 6:**
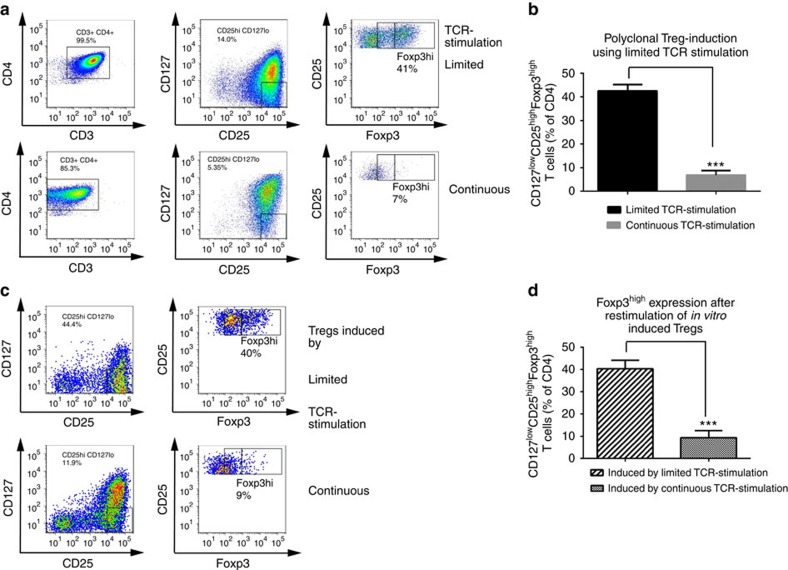
Stability of human Foxp3^+^Tregs induced by sub-immunogenic TCR stimulation *in vitro*. (**a**) Polyclonal induction of Tregs by limited TCR stimulation *in vitro*. Representative FACS plots of limited (12 h) and continuous (54 h) TCR stimulation. (**b**) Frequency of Foxp3^high^ Tregs induced by limited or continuous TCR stimulation. Data are presented as the mean±s.e.m. (*n*=5) of duplicate wells in five individual experiments. ***P<0.001 (Student's *t*-test). (**c**) Stability of Tregs induced by limited or continuous TCR stimulation *in vitro*. Representative FACS plots prepared after re-stimulation of CD127^low^CD25^high^Tregs that had been previously induced by continuous or limited polyclonal TCR stimulation to assess Treg stability. (**d**) Frequency of induced Tregs following re-stimulation. Data are presented as the mean±s.e.m. (*n*=5) of duplicate wells in five individual experiments. ***P<0.001 (Student's *t*-test).

Next, we characterized human Treg stability upon their induction using sub-immunogenic (limited) TCR stimulation^16^,^35^. Sub-immunogenic TCR stimulation significantly increased frequencies of induced CD127^low^CD25^high^Foxp3^high^CD4^+^Tregs compared with non-sub-immunogenic (continuous) TCR stimulation (limited TCR versus continuous TCR stimulation: 42.5±2.7 versus 6.9±1.9 Foxp3^high^Tregs as % of CD127^low^CD25^high^ cells, *P*<0.01, Fig. 6a,b). Importantly, when we re-stimulated the previously induced CD127^low^CD25^high^Tregs, the frequency of Foxp3^high^Tregs was significantly higher when the cells were previously stimulated by limited TCR conditions than cells previously stimulated by continuous TCR conditions (40.3±3.8 versus 9.4±3.2 Foxp3^high^Tregs as % of CD127^low^CD25^high^cells) (Fig. 6c,d). These data support the concept that a subimmunogenic TCR stimulus during human Treg induction *in vitro* confers increased stability of Foxp3^high^Tregs.

### Insulin-specific CD4^+^ T cells in NSG-HLA-DQ8 mice

**Figure 7:**
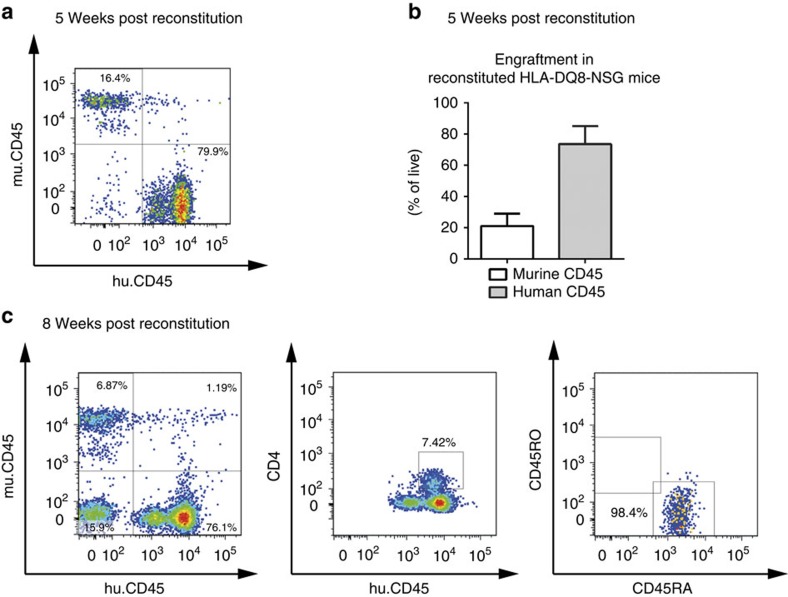
Engraftment efficiency of reconstituted NSG-HLA-DQ8 transgenic mice. (**a**) NSG-HLA-DQ8 mice that had been reconstituted with human HSCs at 2 weeks after birth were first analysed for the engraftment of a human immune system 5 weeks later in peripheral blood. A representative FACS plot is shown to assess engraftment efficiency based on murine versus human CD45 expression levels. (**b**) Summary graphs for identified murine versus human CD45^+^ cells in peripheral blood five weeks after reconstitution; *n*=8 from two independent experiments. (**c**) Representative set of FACS plots 8 weeks post engraftment to confirm human immune cell subsets based on murine versus human CD45 as assessed in peripheral blood. Human CD4 and activation status of human CD4^+^T cells using CD45RA and CD45RO expression.

**Figure 8:**
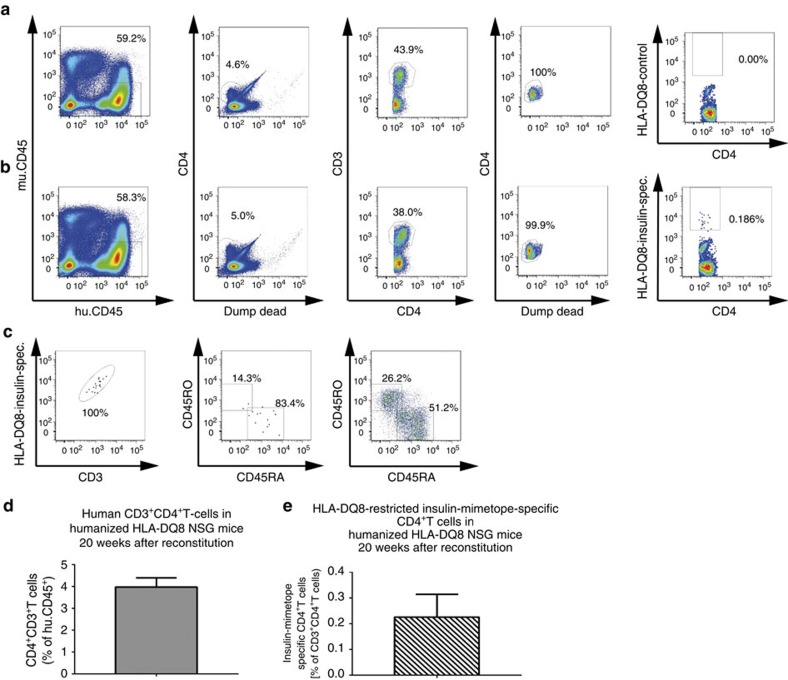
CD4^+^T-cell responses in reconstituted humanized NSG-HLA-DQ8 mice. Human immune subsets purified from pooled spleen and lymph nodes of humanized NSG-HLA-DQ8 mice, 20 weeks post reconstitution, were first identified flow cytometrically based on murine versus human CD45 expression. Human CD4^+^CD3^+^T cells were characterized upon exclusion of dead cells and additional markers (CD8, CD11b, CD14, CD19). (**a-c**) Representative set of FACS plots for the identification of HLA-DQ8-restricted insulin-specific CD4^+^T cells. (**a**) Control staining to assess quality and specificity of the tetramer staining by the use of a combination of two control tetramers fused to irrelevant peptides (**a**; upper row, right plot). (**b**) Representative set of FACS plots for the identification of HLA-DQ8-restricted insulin-specific T cells gating against CD4 (**b**; lower row, right plot). (**c**) Representative set of FACS plots for the phenotypic characterization of identified HLA-DQ8-restricted insulin-specific CD4^+^T cells based on gating against CD3 and CD45RA- versus CD45RO-expression (memory-status, insulin-specific versus polyclonal). (**d**) Summary graphs for identified human CD4^+^T cells purified from spleen and lymph nodes of respective mice, *n*=8 from two independent experiments. (**e**) Summary graph for identified human HLA-DQ8-restricted insulin-specific CD4^+^T cells purified from spleen and lymph nodes, *n*=8 from two independent experiments.

To determine the conditions for human insulin-specific Foxp3^+^Treg induction in the context of a human immune system *in vivo* murine MHCII-deficient NSG-HLA-DQ8 transgenic mice were reconstituted 2 weeks after birth with human HSCs purified from fresh umbilical cord blood from six *HLA-DQ8*^+^ donors. Such reconstituted NSG-HLA-DQ8 mice showed high engraftment efficiency 8 weeks post reconstitution in peripheral blood (human CD45^+^ leukocytes=73.5±5.1%, Fig. 7). When analysed at 20 weeks post reconstitution NSG-HLA-DQ8 mice presented with successful CD4^+^T-cell development in pooled spleen and lymph nodes (3.9±0.4% of human CD45^+^ leukocytes, Fig. 8, *n*=8 from two independent experiments) and peripheral blood (Supplementary Fig. 7) in accordance with previous studies^36^. Upon reconstitution we likewise identified in those animals other immune subsets, for example, human CD8^+^T cells and B cells (Supplementary Fig. 8).

To characterize insulin mimetope-specific CD4^+^T cells we used insulin-HLA-DQ8-tetramers. Of note, we identified HLA-DQ8-restricted insulin mimetope-specific CD4^+^T cells indicating successful positive selection on human HLA-DQ8 molecules in those humanized mice (0.2±0.2 of human CD4^+^T cells, Fig. 8). Expression levels of tetramer^+^CD4^+^T cells correlated with CD3. No tetramer^+^CD4^+^T cells were detected with the control tetramers. Two-third of the insulin-specific CD4^+^T-cell fraction were in a naive CD45RA^+^state (Fig. 8c, middle plot) therefore suitable for Foxp3^+^Treg induction. HLA-DQ8-restricted insulin-specific CD4^+^T cells were likewise identified in peripheral blood but not in CD4^+^T cells purified from white adipose tissues (WATs) (Supplementary Fig. 9).

### Human insulin-specific Foxp3^+^Treg induction *in vivo*

To determine human insulin-specific Foxp3^+^Treg induction *in vivo* using subimmunogenic TCR stimulation reconstituted NSG-HLA-DQ8 mice were subcutaneously implanted with osmotic mini-pumps infusing minute amounts of insulin mimetopes (5 μg per day for 14 days). Based on optimal CD4^+^T-cell development vaccination was done at 20 weeks post reconstitution. In accordance with their enhanced stimulatory potential as identified in insulin-specific CD4^+^T-cell clones (Figs 1 and 4) and optimal *in vitro* Treg induction (Fig. 5) we chose a combination of ins.mim.1=14E-21G-22E and ins.mim.4=14E-21E-22E for *in vivo* Foxp3^+^Treg induction.

**Figure 9:**
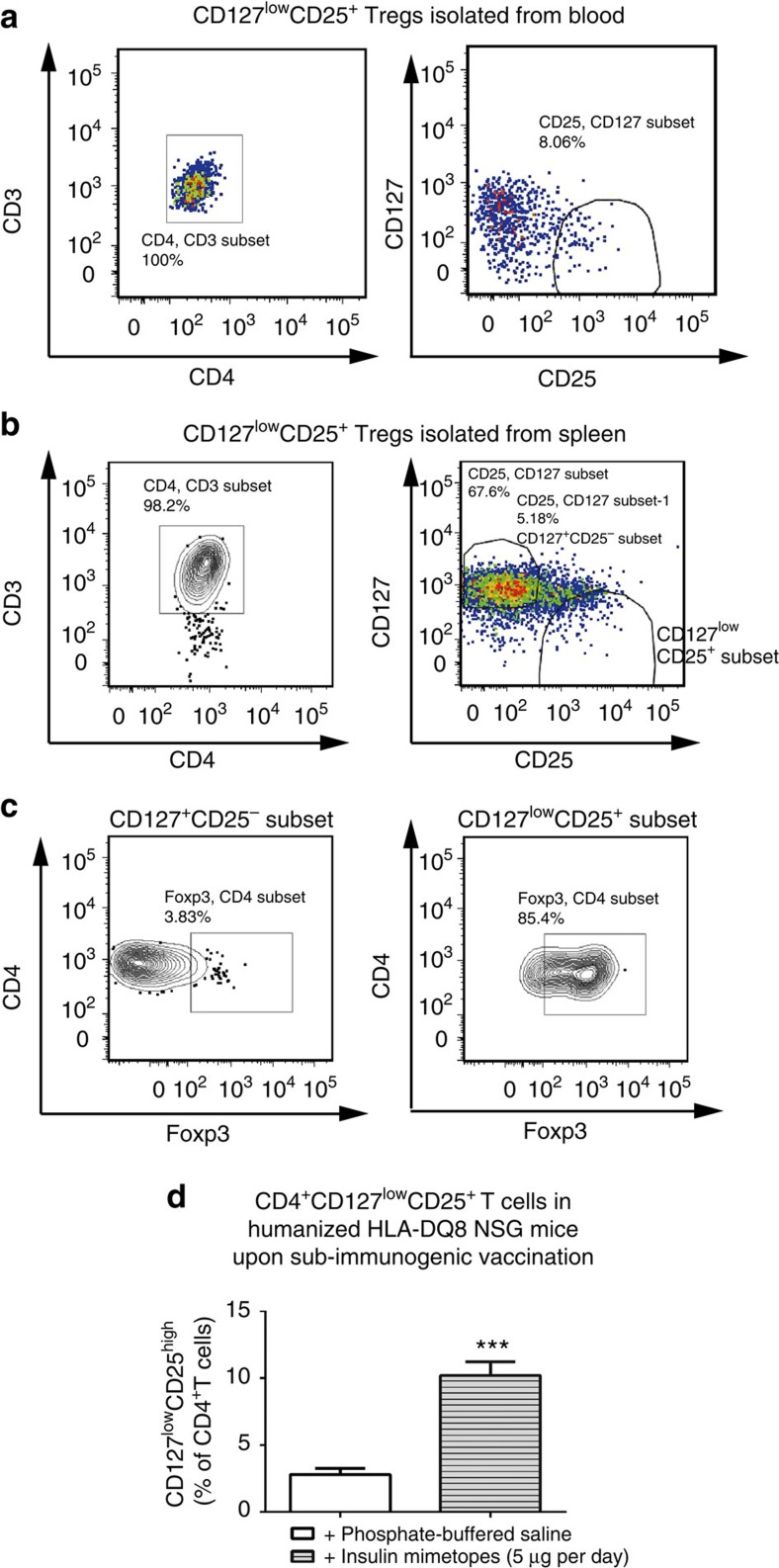
Identification of CD127^low^CD25^+^Tregs in humanized NSG-HLA-DQ8 mice. Human immune subsets isolated from peripheral blood or spleen purified from humanized NSG-HLA-DQ8 mice, 23 weeks post reconstitution, were first identified flow cytometrically based on human CD45 expression. Human CD4^+^CD3^+^T cells were characterized upon exclusion of dead cells and additional markers (CD8, CD11b, CD14 and CD19). (**a**) Representative set of FACS plots for the identification of CD4^+^CD3^+^CD127^low^CD25^+^Tregs isolated from blood. (**b**) Representative set of FACS plots identifying CD4^+^CD3^+^CD127^low^CD25^+^Tregs purified from spleen. (**c**) Verification of Treg phenotype by intracellular staining for Foxp3 in the CD127^+^CD25^-^ non-Treg-subset and the CD127^low^CD25^+^Treg subset as identified in **b** (right plot). (**d**) Summary graphs for the quantification of identified CD127^low^CD25^+^Tregs purified from peripheral blood and spleen upon subimmunogenic Treg induction *in vivo* using insulin mimetopes by osmotic mini-pumps (ins.mim.1=14E-21G-22E; ins.mim.4=14E-21E-22E) or control (PBS) in humanized NSG-HLA-DQ8 mice, *n*=8 from two independent experiments. ***P<0.001 (Student's *t*-test).

Human CD127^low^CD25^+^Tregs were identified in humanized NSG-HLA-DQ8 mice in peripheral blood and spleen (Fig. 9a,b). Treg identity within CD127^low^CD25^+^T cells was verified by intracellular staining for Foxp3 (Fig. 9c).

Three weeks after subimmunogenic vaccination with insulin mimetopes humanized NSG-HLA-DQ8 mice showed significantly increased frequencies of human CD127^low^CD25^+^Tregs (Fig. 9d,+PBS: CD127^low^CD25^+^ Tregs: 2.8±0.4% versus+insulin mimetopes: CD127^low^CD25^+^ Tregs: 10.2±1.0%; *P*<0.001). Upon application of insulin mimetopes we also identified HLA-DQ8-restricted insulin-specific CD127^low^CD25^high^Tregs (Supplementary Fig. 10).

Moreover, when CD4^+^T cells were isolated from pancreatic islets we identified increased frequencies of CD127^low^CD25^+^Tregs in NSG mice that had received insulin mimetopes for Treg induction in contrast to control animals treated with PBS (Supplementary Fig. 11).

Next, when we analysed Ki67 expression in insulin mimetopes treated NSG mice, we observed a higher proliferative potential of CD127^low^CD25^high^Tregs purified from lymph nodes when compared with peripheral blood and pancreatic islets (Supplementary Fig. 12).

### Signatures of induced Tregs in NSG-HLA-DQ8 mice

**Figure 10:**
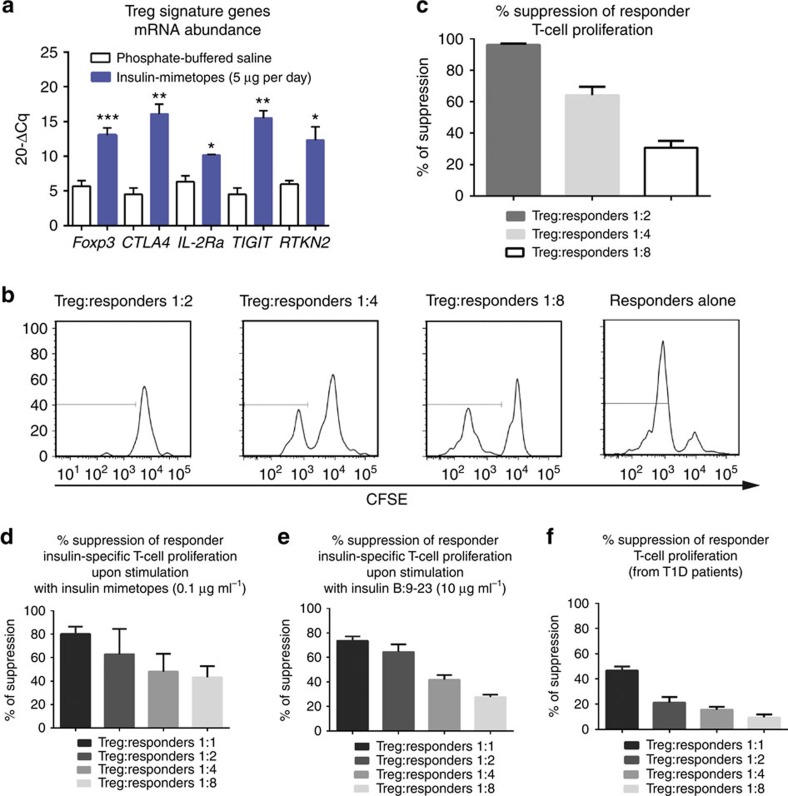
Treg signatures and suppressive potential in humanized NSG-HLA-DQ8 transgenic mice. (**a**) Quantitative PCR with reverse transcription analyses of *Foxp3, CTLA4, IL-2Rα, TIGIT* and *RTKN2* mRNA abundance in human CD4^+^T cells purified from pooled spleens and lymph nodes of humanized mice after 3 weeks of *in vivo* Treg induction using subcutaneous insulin mimetopes infusion by osmotic mini-pumps (ins.mim.1=14E-21G-22E; ins.mim.4=14E-21E-22E) in humanized NSG-HLA-DQ8 transgenic mice (*n*=4). Bars represent the means±s.e.m. (*n*=4 mice per group and experiment, *n*=2 independent experiments). *P<0.05; **P<0.01; ***P<0.001 (Student's *t*-test). (**b**) Analyses of FACS-based suppression assays. Conventional responder CD4^+^T cells or Tregs were purified from pooled spleens and lymph nodes of respective humanized animals. Representative histograms show CFSE dilution profiles of CD4^+^T responder cells alone or in the presence of different ratios of Tregs (1:2; 1:4 and 1:8). (**c**) Summary graphs for the % suppression of responder cell proliferation in the presence of distinct Treg ratios. Values represent means±s.e.m.; *n*=5 mice per experiment, *n*=2 independent experiments). (**d**) Summary graphs for the % suppression of responder cell proliferation using HLA-DQ8-restricted insulin mimetope-specific CD4^+^T-cell clones from children with ongoing islet autoimmunity and stimulation with insulin mimetopes (ins.mim.1=14E-21G-22E; ins.mim.4=14E-21E-22E, final at 0.1 μg ml^−1^) in the presence of distinct Treg ratios. Values represent means±s.e.m.; *n*=5 mice per experiment, *n*=2 independent experiments. (**e**) Summary graphs for the % suppression of responder cell proliferation using HLA-DQ8-restricted insulin mimetope-specific CD4^+^T-cell clones from children with ongoing islet autoimmunity and stimulation with insulin B:9-23 (at 10 μg ml^−1^) in the presence of distinct Treg ratios. Values represent means±s.e.m.; *n*=5 mice per experiment, *n*=2 independent experiments. (**f**) Summary graphs for the % suppression of responder cell proliferation using responder T cells from T1D patients (*n*=3) in the presence of distinct Treg ratios. Values represent means±s.e.m.; *n*=5 mice per experiment, *n*=2 independent experiments.

Upon *in vivo* Foxp3^+^Treg induction insulin-specific CD4^+^T cells purified from spleens of humanized NSG-HLA-DQ8 mice presented with enhanced *Foxp3* abundance as seen from quantitative PCR with reverse transcription analyses thereby further supporting the concept of insulin mimetope-specific tolerance induction (Fig. 10a). Analyses of human Treg signature genes^37^,^38^ revealed enhanced abundance of *CTLA4* and *IL-2Rα* which impact Treg physiology. In addition, we observed significantly increased abundance of *TIGIT* which has been reported as important for Treg suppressive function^39^,^40^ and *RTKN2* which was shown to share the unique Treg signature expression pattern while its functional role in Treg biology remains largely undefined^37^ (Fig. 10a). Upon subimmunogenic vaccination with insulin mimetopes we did not observe any significant changes in *IKZF2* encoding Helios, nor in *ENTPD1* (encoding CD39, a Treg effector molecule^41^). Moreover, no upregulation of T effector cell genes such as *IL-17Rα* and *IL-21* was seen (Supplementary Fig. 13, abundance of *NFATc2, RORγt, T-bet* and *IFNγ* were below the lower limit of detection).

### Stability of human Foxp3^+^Tregs induced *in vivo*

To assess the methylation status of the *Foxp3* CNS2 region (Treg-specific demethylated region (TSDR)) we used high-resolution melting (HRM)-PCR and pyrosequencing (Supplementary Fig. 14). The TSDR region is critically involved in maintaining longterm stability of *Foxp3* expression^42^,^43^. We first evaluated the *Foxp3* TSDR methylation status in *ex vivo* human CD4^+^T cell/Treg populations from male and female donors (Supplementary Fig. 15a,b). Because of the fact that the *Foxp3* gene is X-linked, levels of *Foxp3* TSDR methylation were higher in T cells from female compared with male donors.

Next, we found that upon Foxp3^+^Treg induction *in vivo* human CD127^low^CD25^high^Foxp3^high^Tregs purified from spleens and lymph nodes of humanized NSG-HLA-DQ8 mice presented with a demethylated TSDR region (Supplementary Fig. 15c). The methylation status of such Tregs from humanized mice induced by application of insulin mimetopes was as low as levels seen in *ex vivo* human Foxp3^high^Treg populations (Supplementary Fig. 15). In contrast, the TSDR region from naive CD4^+^T cells of such humanized NSG mice was completely methylated (Supplementary Fig. 15c).

To further assess the stability of human Tregs induced upon subimmunogenic TCR stimulation *in vivo*, humanized NSG-HLA-DQ8 mice were maintained for 6 months upon Treg induction. After 6 months humanized NSG mice that had received insulin mimetopes for Foxp3^+^Treg induction presented with significantly enhanced abundance of *Foxp3, CTLA4, IL-2Rα* and *TIGIT* compared with control animals (Supplementary Fig. 16a). Moreover, 6 months after subimmunogenic vaccination no upregulation of T effector cell genes had occurred (Supplementary Fig. 16b). In accordance, CD4^+^T cells from such humanized NSG mice also harboured reduced abundance of *IL-17Rα, IL-21* and *IFNγ* when compared with control animals, (Supplementary Fig. 16b, abundance of *NFATc2, RORγt* and *T-bet* remained below the lower limit of detection).

These findings are in accordance with data obtained in murine T1D. We purified CD4^+^T cells from pancreatic lymph nodes of 40-week-old NOD Foxp3-GFP reporter mice that had either received the natural insulin-B-chain epitope or insulin mimetope (ins.mim.3=21E-22E) for Treg induction at the age of 4–6 weeks. Using two fluorescent insulin mimetope-specific IA^g7^-tetramers (21G-22E- and 21E-22E-tetramer)^28^ we showed that NOD mice that had received the insulin mimetope and were still diabetes-free presented with significantly increased frequencies of insulin-specific Foxp3^+^Tregs (7.2±1.8 versus 20.2±1.7% of insulin-specific CD4^+^T cells, *P*<0.01, Supplementary Fig. 17).

Moreover, T cells purified directly from the islets of NOD mice that had received insulin mimetopes for Treg induction presented with increased frequencies of stable Foxp3^+^Tregs accompanied by a demethylated *Foxp3* TSDR region (Supplementary Fig. 18).

### Suppressive potential of induced tregs *in vivo*

For further functional analyses of human CD127^low^CD25^high^Tregs purified from humanized mice upon subimmunogenic vaccination we performed *in vitro* suppression assays^44^. Human CD127^low^CD25^high^Tregs and conventional T cells were sort-purified from spleens and lymph nodes and first expanded using polyclonal stimulation. Tregs and conventional T cells were rested for 16 h in the absence of IL-2 to force them into synchronous resting states^44^. Suppression of proliferative responses of conventional CD4^+^T cells was then determined by analysing the dilution of their CFSE label in the presence or absence of Tregs. *Ex vivo* human Tregs presented with potent suppressive capacities (% suppression of responder cell proliferation: Treg: responder 1:2=96.1±0.4; 1:4=64.1±3.8; 1:8=30.8±3.0; Fig. 10b,c).

Next, we performed suppression assays with HLA-DQ8-restricted insulin-specific T-cell clones cloned from children with ongoing islet autoimmunity as responder cells and insulin mimetopes or insulin B:9-23 for stimulation. Induced Tregs from humanized mice suppressed insulin mimetope-specific proliferation (% suppression of insulin-specific responder cells proliferation: Treg: responder 1:1=80.3±3.5; 1:2=62.8±12.7; 1:4=48.2±8.7; 1:8=43.2±5.5; Fig. 10d). Likewise, such Tregs suppressed proliferation of insulin-specific T-cell clones upon stimulation with insulin B:9–23 (% suppression of insulin-specific responder cells proliferation: Treg: responder 1:1=73.5±2.5; 1:2=64.5±4.4; 1:4=41.8±2.7; and 1:8=27.6±1.5; Fig. 10e).

Moreover, such induced Tregs from humanized mice suppressed responder T-cell proliferation using effector T cells from T1D patients (% suppression of responder cells proliferation: Treg: responder 1:1=46.6±2.3; 1:2=21.4±2.4; 1:4=15.6±1.6; and 1:8=9.5±1.7, Fig. 10f).

## Discussion

Control of autoimmunity through instruction of endogenous regulatory mechanisms is a long envisioned challenging goal of physicians and scientists^18^. In man, the development of autoantigen-specific tolerance induction strategies is still in its infancy and currently studies are ongoing. Initial results from clinical trials using natural autoantigens for induction of self-tolerance, for example, natural insulin in T1D showed thus far little benefit^45^,^46^,^47^,^48^. More recently, a first primary insulin-specific vaccination dose-finding study in children genetically susceptible to T1D was finished^49^ where oral insulin application to children genetically at risk but without ongoing islet autoimmunity supported an immune response.

To further advance the translation of these antigen-specific therapies from bench to bedside it will be critical to investigate whether the choice of antigen, the time point and route of administration induced a tolerogenic response and specifically to study the conditions for efficient human Foxp3^+^Treg induction. It has been suggested that efficacy of tolerance induction may critically depend upon: disease state, antigen dosage, route of administration, the study cohort that is treated and the choice of antigen, for example, insulin versus insulin B chain peptides.

In humans, T1D risk is linked strongly to combinations of the *HLA-DR4/DQ8* and *DR3/DQ2* haplotypes^50^ with 90% of T1D patients harbouring *DQ8* or *DQ2* alleles. HLA-DQ8 shares with I-A^g7^ strikingly similar binding pockets for peptide presentation^29^. Insulin B:9-23-reactive CD4^+^T cells are present in the peripheral blood of T1D individuals, the immunogenic register of this peptide has low-affinity binding to HLA-DQ8 (ref. 30) and a strong agonistic variant of the natural insulin epitope established in the murine system^7^ can efficiently stimulate human CD4^+^T cells^31^.

Here, we identify two novel human insulin mimetopes (ins.mim.1=14E-21G-22E and ins.mim.4=14E-21E-22E) with increased stimulatory capacities when compared with the natural insulin B:9-23 epitope and previously established insulin variants^7^,^17^,^30^,^31^. The combination of ins.mim.1 and ins.mim.4 was chosen to use as the best stimulating mimetic within each category of insulin-reactive CD4^+^T cells, namely type A and type B cells, as suggested in the NOD mouse setting^28^. However, based on its highest affinity to HLA-DQ8 ins.mim.4 (14E-21E-22E) will probably contribute most to the observed functional effects.

Tetramers using ins.mim.1 and ins.mim.4 permitted for the first time the direct *ex vivo* identification of human insulin mimetope-specific CD4^+^T cells without prior *in vitro* expansion^30^ and combined with intracellular Foxp3 staining the immediate analysis of insulin mimetope-specific Foxp3^+^Tregs. We have demonstrated that high frequencies of insulin mimetope-specific Tregs were associated with profound delays in T1D progression in children, which supports the rationale for inducing insulin-specific Foxp3^+^ Tregs to delay or even prevent human T1D. Moreover, these data are consistent with the observation that children with slowly progressing phenotypes display an accumulation of protective genotypes in T1D susceptibility genes^51^ most notably *IL-2, IL2-Rα, INS VNTR* and *IL-10*.

The ability to directly identify human insulin-specific Foxp3^+^Tregs *ex vivo* will be of critical relevance to assess insulin-specific vaccination responses. This is a critical step currently missing in clinical T1D prevention efforts and will support the development of novel T cell-specific biomarkers alongside personalized strategies for efficient prevention of islet autoimmunity and T1D.

The best stimulating human insulin mimetopes when applied at low subimmunogenic doses were also most efficient in inducing human insulin-specific Foxp3^+^Tregs. These results are in accord with observations in NOD mice where subimmunogenic doses of strongly stimulating insulin mimetopes efficiently induced insulin-specific Foxp3^+^Tregs which prevented T1D development^17^. Here, we provide novel conceptual evidence for using low doses of strong-agonistic insulin mimetopes for efficient human Foxp3^+^Treg induction and suppression of human autoimmunity.

With respect to safety aspects of insulin-specific vaccination strategies, recently a first primary insulin-specific vaccination dose-finding study in children genetically susceptible to T1D was completed^49^. Application of high doses of insulin to genetically at-risk healthy children without signs of islet autoimmunity promoted an immune response without hypoglycemia. The incidence and type of adverse events were not different between children who received placebo and children who received insulin, regardless of the insulin dose^49^.

Moreover, insulin peptides have also proved safe at early stages of clinical development, supporting the concept for epitope-based vaccines^52^.

Concerning the time point of vaccination for Treg induction, we show that this process was most efficient in naive T cells from children without ongoing autoimmunity or in non-diabetic children with long-term autoimmunity. It is therefore suggested that insulin mimetope-specific Foxp3^+^Treg induction may be better applied as a primary preventive approach or as a secondary vaccination strategy for non-diabetic children with longer autoimmunity that have successfully passed the critical period of autoimmune development without progression to overt disease (longterm autoimmunity without T1D).

Mechanistically, recent data highlight a critical impact of peptide-MHC quality (stimulation by a strong-agonistic ligand) versus quantity on *in vivo* T-cell responses^53^. Evidence for ligand discrimination beyond sensing of a cumulative TCR signal in that T cell responses differed between low-density and low-potency weak stimuli^17^,^22^,^53^,^54^,^55^ was provided. These findings underline the importance of integrating peptide-MHC quality and quantity in determining the minimal TCR stimulation required for T-cell proliferation *in vivo*^53^ and support our observations that most efficient stable Foxp3^+^Treg induction is achieved by a subimmunogenic stimulus of a strong-agonistic ligand^17^. Accordingly, Tregs induced in humanized NSG mice presented with a demethylated TSDR region and were maintained for prolonged periods of time in the absence of effector cell responses.

More studies are required to gain an improved understanding of how the subimmunogenic application of antigens for the efficient and stable induction of Foxp3^+^Treg cells can be best achieved in human autoimmune diseases. These efforts might include novel strategies for the application of self-antigens—for example, the use of dissolving microneedle patches^56^, which were recently tested for the administration of insulin to individuals with T1D^57^. Such novel application strategies could help to mimic continuous subimmunogenic antigen application promoting efficient Foxp3^+^Treg induction. Safety and efficacy of such novel devices for vaccination have been recently tested on human skin^58^,^59^.

It has been shown that human HSC-engrafted NSG mice harbour a highly-diverse TCR repertoire, which is critical for mounting an efficient yet not self-destructive adaptive immune response^60^. The replacement of mouse MHC molecules by human MHC components has been a major advance in increasing the utility of these ‘humanized' mice as this permits the generation and maintenance of robust human T cell responses^61^.

In reconstituted NSG-HLA-DQ8 mice we provide first direct evidence for HLA-DQ8-restricted insulin-specific CD4^+^T-cell responses indicating positive selection on human HLA-DQ8 molecules. Moreover, this will allow studying the requirements for human insulin-specific Foxp3^+^Treg induction in further detail.

In sum, in the pre-clinical setting of humanized NSG-HLA-DQ8 transgenic mice we established for the first time subimmunogenic Foxp3^+^Treg induction and demonstrate that subimmunogenic application of insulin mimetopes promotes enhanced levels of insulin-specific Foxp3^+^Tregs in a human immune system *in viv*o. Moreover, such induced Tregs were found to be stable and presented with robust suppressive capacities and increased abundance of Treg signature genes.

It remains challenging to interfere with processes that generate autoimmune T1D in patients; however, defining the requirements for efficient human insulin-specific Foxp3^+^Treg induction *in vivo* as evidenced here by the establishment of subimmunogenic vaccination protocols in humanized mice alongside the development of novel human insulin mimetopes could represent a critical improvement in this challenge.

## Methods

### Human subjects and blood samples

For the present study blood samples were collected from children or adults who are first degree relatives of patients with T1D. Written consent was obtained for the *Munich Bioresource project (approval number #5049/11, approval committee: Technische Universität München, Munich, Germany)*. All subjects have been already enrolled into longitudinal studies with prospective follow-up from birth^62^,^63^,^64^ with the documented age of islet autoantibody seroconversion (initiation of islet autoimmunity). Venous blood was collected using sodium heparin tubes and blood volumes collected were based on EU guidelines with a maximal blood volume of 2.4 ml kg^−1^ of body weight. Subjects have been stratified based on the presence or absence of multiple islet autoantibodies (=with or without pre-T1D) and based on the duration of islet autoantibody positivity: no autoimmunity: first degree relatives of patients with T1D who are islet autoantibody negative (*n*=11; median age=8 years, interquartile range (IQR)=6–12 years, six males, five females); recent onset of islet autoimmunity: subjects with multiple islet autoantibodies for less than 5 years (*n*=7, median age=5 years, IQR=4–14 years, five males, two females); persistent autoimmunity: subjects with multiple islet autoantibodies for more than 5 but less than 10 years (*n*=9, median age=14 years, IQR=10–17.5 years, 7 males, two females); longterm autoimmunity: subjects with multiple islet autoantibodies for more than a decade who did not yet develop T1D (*n*=7, median age=15 years, IQR=14–25 years, three males, four females). In addition five children with newly diagnosed T1D and very early disease manifestation (disease onset<5 years) were studied (*n*=5, median age=4 years, IQR=2–8 years, two males, three females) and six children with longterm T1D for >5 years have been included (*n*=6, median age=14, IQR=11.5–16.5 years, five males, one female). Umbilical cord blood from healthy full-term newborns, acquired immediately after delivery from the clamped umbilical cord was collected in citrate phosphate dextrose, including one from a child of a mother with T1D. Samples (*n*=6) were provided through the DKMS Cord Blood Bank of the University Hospital Dresden (Germany) or from the Institute of Diabetes Research, Klinikum rechts der Isar, Technische Universität München with informed consent and local ethics committee approval (approval number: #5293/12, Technische Universität München, Munich, Germany).

### Mice

NOD.129X1(Cg)-*Foxp3*^*tm2Tch*^/DvsJ mice, referred to as NOD Foxp3 GFP reporter mice, were obtained from The Jackson Laboratory. Antigen-specific *in vivo* Treg cell conversion protocols were executed based on established protocols^17^: Four weeks-old female NOD Foxp3 GFP reporter mice were implanted subcutaneously with osmotic mini-pumps (Alzet) releasing 5 μg day^−1^ of insulin mimetopes or the natural insulin-B-chain epitope for 14 days. Mice were randomized to test groups for antigen-specific Treg conversion. No animals were excluded due to illness or outlier results; therefore, no exclusion determination was required. For *ex vivo* analyses of induced insulin-specific Foxp3 GFP^+^ Tregs, the entire group of mice for treatment with either the natural insulin-epitope or the strong-agonistic mimetope was analysed. NOD.Cg-*Prkdc*^*scid*^ *H2-Ab1*^*tm1Gru*^ *Il2rg*^tm1Wjl^ Tg(HLA-DQA1,HLA-DQB1)1Dv//Sz mice lack mouse MHC class II and transgenically express human *HLA-DQ8*. These mice were developed by Leonard Shultz at the Jackson Laboratory. To develop this stock, B10M-HLA-DQ8 mice were kindly provided by Dr. Chella David^65^. The DQ8 transgene was backcrossed for 10 generations on the NSG strain background. The NSG-DQ8 mice were then intercrossed with NSG mice lacking mouse MHC class II (NOD.Cg-*Prkdc*^*scid*^ *H2-Ab1*^*tm1Gru*^ *Il2rg*^tm1Wjl^) (ref. 66). The HLA-DQ8 mice were bred and maintained group-housed on a 12-h/12-h light dark cycle at 25 °C with free access to food and water under defined flora at the animal facility of Helmholtz Zentrum München, Munich, Germany and at The Jackson Laboratory according to guidelines established by the Institutional Animal Committees at each institution. These mice were used as hosts for human HSC obtained from human HLA-DQ8 cord blood samples. The sex of the recipient mice was matched for the HSC donor sex. Ethical approval for all mouse experimentations has been received by the District Government of Upper Bavaria, Munich, Germany (approval numbers: #55.2-1-54-2532-81-12 and 55.2-1-54-2532-84-12). The investigators were not blinded to group allocation during the *in vivo* experiments or to the assessment of experimental end points.

### Isolation of infiltrating T cells from murine pancreata

Pancreata were digested with collagenase V (1 mg ml^−1^) in PBS with 0.1 mM HEPES and 0.1% BSA for 4–7 min at 37 °C. The cell suspension was passed through a 100 μm cell strainer and stained for flow-cytometric analysis.

### Human cell isolation

Peripheral blood mononuclear cells (PBMC) were isolated by density centrifugation over Ficoll-Paque PLUS (GE Healthcare). HSCs were purified from PBMCs from fresh umbilical cord blood using the CD34^+^ isolation kit (Diamond CD34 Isolation kit human, Miltenyi Biotec) according to the manufacturer's protocols. Human Dendritic cells (DCs) were purified from autologous PBMC samples using the Blood DC Isolation Kit (Blood DC Isolation kit II human, Miltenyi Biotec) according to the manufacturer's instructions. Specifically CD14^+^ and CD19^+^ cells were labelled with magnetic beads and depleted from the PBMC sample by separation on a MACS column. Subsequently the remaining cells were labelled with CD304, CD1c and CD414 magnetic beads and positive selection over a MACS column of CD304^+^ plasmacytoid and CD1c^+^ and CD141^+^ myeloid DCs was performed. Human CD4^+^T cells were isolated from fresh PBMCs via negative magnetic bead enrichment (EasySep Human CD4 T Cell enrichment kit, Stem Cell) following the manufacturer's protocol.

### Cell staining for flow cytometry and cell sorting

The following monoclonal antibodies were used for murine fluorescence-activated cell sorting (FACS) staining: From Biolegend (San Diego, CA): anti-CD8a Pacific Blue (53–6.7, 1:300); anti-CD11b Pacific Blue (M1/70; 1:300), anti-CD11c Brilliant Violett 421 (N418, 1:300); anti-B220 Pacific Blue (RA3-6B2, 1:300), anti-F4/80 Pacific Blue (BM8, 1:300), anti-CD25 PerCP-Cy5.5 (PC61, 1:200), anti-CD44 PE (IM7, 1:800), anti-CD45 PE-Cy7 (30-F11, 1:200) and Ki67 APC (16A8, 1:200); and from eBioscience (San Diego, CA): anti-CD4 Alexa Fluor 700 (RM4-5; 1:200), anti-CD62L APC (MEL-14, 1:400) and Foxp3 FITC (FJK-16 s, 1:200). Enumeration of cells and acquisition were performed by using FACSAria and FACSDiva software (BD). Single-cell data analyses are done by the use of the FlowJo software (Tree Star Inc.).

The following monoclonal antibodies were used for human FACS staining: from BD Biosciences (San Jose, CA): anti-CD25 APC (2A3, 1:20), anti-CD45RO APC-H7 (UCHL1, 1:20), anti-CD4 V500 (RPA-T4, 1:20) and anti-HLA-DR PerCP-Cy5.5 (L243, 1:20); from Biolegend (San Diego, CA): anti-CD45RA FITC (HI100, 1:20), anti-CD3 PerCP-Cy5.5 (HIT3a, 1:20), anti-CD127 PE-Cy7 (A019D5, 1:20), anti-CD8a Pacific Blue (RPA-T8, 1:50), anti-CD11b Pacific Blue (ICRF44, 1:50), anti-CD14 Pacific Blue (HCD14, 1:50), anti-CD19 Pacific Blue or Alexa Fluor 700 (HIB19, 1:50), anti-CD3 Alexa Fluor 700 (HIT3a, 1:20), anti-CD45 Alexa Fluor 700 (HI30, 1:20), anti-CD34 Brilliant Violet 421 or APC (561, 1:20), anti-CD38 PE (HIT2, 1:20), anti-C-kit PE-Cy7 (104D2, 1:20), anti-lineage cocktail (CD3, CD14, CD16, CD19, CD20, CD56) APC or FITC (UCHT1, HCD14, 3G8, HIB19, 2H7, HCD56, 1:5) anti-CD14 (HCD14), anti-CD33 V500 (WM53, 1:20), anti-Ki67 APC (16A8, 1:200) or anti-Ki67 Brilliant Violet 605 (16A8, 1:400); from eBioscience (San Diego, CA): anti-Foxp3 Alexa Fluor 700 (PCH101, 1:100), anti-Foxp3 PE (236A/E7, 1:100); and from Miltenyi Biotech: anti-CD20 PE (2H7, 1:5).

To detect intracellular protein expression of Foxp3, after surface staining, cells were fixed and permeabilized using the Foxp3 Staining Buffer Set (eBioscience). Cells were acquired on a Becton Dickinson LSR-II or on the BD FACSAria III cell sorting system flow cytometer using FACSDiva software with optimal compensation and gain settings determined for each experiment based on unstained and single-colour stained samples. Doublets were excluded based on SSC-A versus SSC-W plots. Live cell populations were gated on the basis of cell side and forward scatter and the exclusion of cells positive for 7-AAD (BD Biosciences) for murine stainings and Sytox Blue (Life Technologies) or Fixable Viability Dye eFluor450 (eBioscience) for human stainings. Samples were analysed using FlowJo software version 7.6.1 (TreeStar Inc., OR).

### Peptides

Peptides at >95% purity were synthesized and purified at New England Peptide (Boston, USA) or at JPT Peptides (Berlin, Germany). Peptide sequences. HA_307-319_ epitope: H2N-PKYVKQNTLKLAT-OH, natural insulin B:9-23 epitope: H2N-SHLVEALVLVCGERG-OH, four insulin-B-chain-10-23-mimetopes (Supplementary Fig. 1) were employed in studies using human CD4^+^T cells. Peptides were chosen first based on the finding that insulin-B:10-23 peptide variants with a mutation of arginine (R) to glutamic acid (E) at position 22 and/or including a change to glycine (G) at position 21 (ins.mim.2=21G-22E; ins.mim.3=21E-22E) were indicated to be more potent in stimulating murine insulin-specific CD4^+^T cells^7^,^17^,^28^ and also suited to stimulate human insulin-specific CD4^+^T cells^30^,^31^.

Second, two novel human insulin mimetopes with mutations at position 22 to glutamic acid (E) together with position 21 being E or G as well as an additional mutation of position 14 from alanine (A) to glutamic acid (E) (ins.mim.1=14E-21G-22E; ins.mim.4=14E-21E-22E) were set up. The mutation at position 14 was included since structural analyses of a human insulin-peptide-HLA-DQ8 complex had suggested that glutamic acid (=E) is preferred over alanine at the first MHC-anchor^29^.

### Insulin-specific IA^g7^-restricted tetramer staining

Tetramer stainings have been performed using established insulin mimetope-specific 22E- and 21G-22E-tetramers^28^. In brief, untouched CD4^+^T cells were incubated with tetramer reagents for 1 hour at 37 °C in humidified 5% CO_2_ with gentle agitation every 30 min followed by direct staining with antibodies for additional surface markers and exclusion of dead cells for 20 min at 4 °C. A set of exclusion markers (CD8, CD11b, CD11c, B220, F4/80 and a dead cell exclusion marker (Sytox Blue)) was used to increase specificity of the staining. As a negative control, we used a tetramer of IA^g7^ with the well-characterized peptide from hen egg lysozyme labelled with PE.

### Insulin-specific HLA-DQ8-restricted tetramer staining

Fluorescent HLA-DQ8-tetramers based on insulin-B-chain-10-23-mimetopes were developed in collaboration with the NIH tetramer facility. Specifically, two of the insulin-HLA-DQ8-PE-labelled tetramers were combined in stainings: a 14E-21E-22E and a 14E-21G-22E-tetramer were used to identify human insulin-specific CD4^+^T cells. For the HLA-DQ8-restricted insulin-specific tetramer stainings PBMCs were used and CD4^+^T cells were purified by negative MACS selection as described above. To this end, untouched CD4^+^T cells were incubated with insulin-specific HLA-DQ8-tetramers for 1 hour at 37 °C in humidified 5% CO_2_ with gentle agitation every 20 min followed by direct staining with antibodies for additional surface markers and exclusion of dead cells (Sytox Blue) for 20 min at 4 °C. A set of exclusion markers (CD8, CD11b, CD19, CD14 and a dead cell exclusion marker (Sytox Blue)) was used to increase specificity of the staining. As negative controls, we used a combination of two HLA-DQ8-tetramers fused to irrelevant peptides (PVSKMRMATPLLMQA and QDLELSWNLNGLQADL) and labelled with PE. Virtually no tetramer^+^CD4^+^T cells were detected with the control tetramers. Upon exclusion of unspecific binding, viable CD3^+^CD4^+^tetramer^+^T cells were single-cell sorted for T-cell cloning experiments, expansion, testing of antigen-specificity or used in further downstream assays.

### HLA-DQ8-binding assay

Competitive binding assays were carried out according to previously established procedures^30^,^67^,^68^: HLA-DQ8 monomers were kindly provided by R.A.W. from the NIH Tetramer Core Facility (Atlanta, USA). The CLIP peptide of HLA-DQ8 molecules was cleaved off by incubation with thrombin (Novagen) for 2 h (ref. 69).

Specifically, a FITC-labelled GAD65 253-265^R255F^ peptide (IAFFKMFPEVKEK) was used as an indicator peptide (10 μM) for the binding reaction together with thrombin-cleaved HLA-DQ8 monomers (0.4 μM) and increasing concentrations of competitor peptides (natural insulin B:9-23, ins.mim.1,2,3,4, MP185-204). The MP185-204 peptide (TAKAMEQMAGSSEQAAEAME) was used as a positive DQ8-binding control. The indicator peptide incubated with DQ8 monomers in the absence of competitor peptide was used as positive control. For background analysis the binding reaction was performed without HLA-DQ8 monomers. The binding reaction was incubated for 48 h at 37 °C. Assays were then captured using anti-DQ antibody-coated plates (SPV-L3, Abcam, 15 μg ml^−1^). Detection was performed using anti-FITC HRP (Abcam, 1:1,000) antibodies in combination with TMB substrate (BD Biosciences) and subsequent analysis with the Epoch plate reader (Biotech) at 450 and 405 nm.

Binding curves were fitted by nonlinear regression using log transformed *x* values (*x*=test peptide concentration) with the one-site competitive binding model to extract IC_50_ values (Prism software, v.6.04, GraphPad Software).

### Generation of artificial antigen-presenting cells

Earlier studies had shown that an indirect coating of fluorescently unlabelled HLA-peptide tetramers on beads via an anti-MHCII antibody provides specific and efficient stimulation of antigen-specific CD4^+^ T cells^34^. Therefore, we first coated anti-HLA-DQ antibodies (SPV-L3, Abcam) to antibody-coupling beads (Dynabeads Antibody Coupling Kit, Life Technologies) at 20 μg mg^−1^ beads followed by coupling with unlabelled HLA-DQ8-tetramers (3 μg per 10 × 10^6^ beads) to the DQ-antibodies. Artificial APCs (aAPCs) using the above described control tetramers were generated accordingly. For stimulation aAPCs were used at a concentration of 230 μg ml^−1^ corresponding to a tetramer concentration of 5 μg ml^−1^.

### CFSE-T-cell proliferation assays

CD4^+^CD25^-^T cells were labelled with CFSE and incubated with propagated APCs loaded with medium alone, various doses of insulin B:9-23 peptide, or with a titration of various strong-agonistic insulin mimetopes (as described above) for 5 days. In all assays, each condition was performed in triplicate wells. Cells were cultured in X-Vivo15 Medium supplemented with 2 mM glutamine, penicillin (50 U ml^−1^), streptomycin (50 μg ml^−1^) and 5% heat-inactivated human AB serum (Invitrogen) in round bottom 96-well plates. After 5 days, the cell cultures were stained for CD4, CD3, CD25 and CD45RO and processed for FACS analyses. Responsiveness was measured by the presence and quantity of CD4^+^CD25^+^CFSE^dim^T cells identified by FACS.

### Generation of insulin-specific T-cell clones

To perform further phenotyping of insulin-specific CD4^+^T cells and to generate specific T-cell clones 500.000 CFSE-labelled CD4^+^T cells were cultured in the presence of insulin-specific aAPCs or control aAPCs generated as described above for 7 days. At day 7 the cells were analysed and a single viable CFSE^dim^CD4^+^T cell was sorted into each well of a 96-well plate in the presence of 200 μl of X-Vivo15 medium and 1 × 10^4^ PBMCs of a *HLA-DQ8*^*−*^ donor, 1 × 10^4^ PBMCs of a *HLA-DQ8*^*+*^ donor as well as 1 × 10^4^ *HLA-DQ8*^*+*^ EBV-transformed B cells (Riken Cell Bank, Japan). Feeder cells were irradiated with 40 Gy (PBMCs) or 50 Gy (B cells) before addition to the cultures. Cells were stimulated with 30 ng ml^−1^ anti-CD3 (OKT3, BioLegend) in the presence of IL-2 (Peprotech, 20 U ml^−1^) and IL-4 (Peprotech, 10 ng ml^−1^). Expansion of specific clones was performed by addition of IL-15 (Peprotech, 10 ng ml^−1^) and IL-21 (Peprotech, 10 ng ml^−1^) as well as low-dose IL-7 (Peprotech, 0.1 ng ml^−1^). For expansion of growing clones cells were splitted into 48-well plates after 2 weeks. Clones were re-tested for antigen-specificity and DQ-restriction by stimulation with natural insulin-peptides or -mimetopes (0.1–10 μg ml^−1^) in the presence or absence of HLA-DQ blocking antibodies (SPV-L3, Abcam, 10 μg ml^−1^) and analysis of total CD25 upregulation and CD25^+++^ levels after 48 h of stimulation was assessed by FACS analyses.

### Analysis of stimulatory potential of insulin mimetopes

Proliferative responses of HLA-DQ8-restricted insulin-specific CFSE-labelled CD4^+^T-cell clones were defined using a titration of individual insulin mimetopes and the natural insulin epitope B:9-23 presented by irradiated T cell depleted PBMCs. Subsequent FACS analyses of CFSE^dim^CD25^+^CD4^+^T cells were performed as described above. The stimulatory capacity of the insulin mimetopes was assessed as fold of stimulatory capacity of the natural insulin B:9-23 epitope.

### Human Treg induction using limited TCR-stimulation *in vitro*

For polyclonal Treg induction, human naive CD4^+^T cells were defined as CD3^+^, CD4^+^, CD45RA^+^, CD45RO^−^, CD127^+^, CD25^−^, HLA-DR^−^ and sorted with the BD FACSAria III for purity (see Supplementary Fig. 6). CD4^+^T cells were cultured for 12 h in a 96-well plate pre-coated with 5 μg ml^−1^ anti-CD3 (UCHT1, BioLegend) and 5 μg ml^−1^ anti-CD28 (CD28.2, BioLegend) and 100 U ml^−1^ IL-2 (Peprotech). Limited TCR stimulation was achieved by pipetting the cells into new, uncoated wells, after 12 h, where they were cultured for additional 36 h without further TCR stimulation. To assess Treg induction using continuous TCR stimulation naive CD4^+^T cells were stimulated in pre-coated wells as described above for a time period of 54 h and analysed accordingly. For antigen-specific Treg induction, human naive CD4^+^T cells were defined as CD3^+^, CD4^+^, CD45RA^+^, CD45RO^−^, CD127^+^, CD25^-^, HLA-DR^−^ and sorted with the BD FACSAria III for purity. Naive CD4^+^T cells were co-cultured with autologous CFSE-labelled DCs isolated as described above in the presence of insulin mimetopes, natural insulin B:9-23 epitope (0.001 and 0.01 ng ml^−1^). After 12 h APCs were removed by sorting CD4^+^T cells as CFSE^−^ followed by a culture for additional 36 h in new wells without further peptide stimulation.

### Restimulation cultures

Upon Treg induction using limited or continuous TCR-stimulation *in vitro* sort-purification of CD127^low^CD25^high^CD4^+^ Tregs was performed. The Tregs were then stimulated for 36 h in the presence 5 μg ml^−1^ anti-CD3 (UCHT1, BioLegend) and 5 μg ml^−1^ anti-CD28 (CD28.2, BioLegend) antibodies without addition of TGFβ followed by the analysis of CD25, CD127 and Foxp3 by intracellular staining and FACS as described above.

### Treg suppression assay *in vitro*

Tregs were sort-purified as CD4^+^CD3^+^CD127^low^CD25^high^T cells. In control experiments Treg identity of CD4^+^CD3^+^CD127^low^CD25^high^ T cells was confirmed by intracellular staining for Foxp3. Conventional T cells were sorted as CD4^+^CD3^+^CD127^+^CD25^−^. Tregs were purified from spleens and lymph nodes of humanized mice and first expanded for six days by polyclonal stimulation with anti-CD3 (UCHT1, BioLegend) and anti-CD28 (CD28.2, BioLegend) at 1 μg ml^−1^ each in the presence of IL-2 (Peprotech, 500 U ml^−1^) and 20 × 10^4^ irradiated CD4^−^ feeder cells (CD4-depleted PBMCs and EBV-transformed B cells). On day six Tregs and conventional T cells were sort-purified to remove the remaining feeder cells and conventional T cells (responder cells) were labelled with CFSE (0.25 μM). Conventional T cells were expanded accordingly at 50 U ml^−1^ Il-2. Treg cells and conventional T cells were rested for 16 h in the absence of IL-2 to force them into synchronous resting states^44^. Labelled responder T cells were cultured with or without Tregs (responder: Tregs 1:2; 1:4 and 1:8) for 3 days in the presence of stimulation with anti-CD3 (UCHT1, BioLegend) and anti-CD28 (CD28.2, BioLegend) (1 μg ml^−1^ each). Analyses were performed on day three on a FACSAria III and suppression of responder cell proliferation was assessed by determining the dilution of their CFSE label. Suppression of responder cell proliferation is shown in % suppression of the proliferation of the responder cells alone^44^.

For insulin-specific suppression assays, induced Tregs from humanized mice were sort-purified as indicated above. Cells of insulin-specific T-cell clones were used as effector cells labelled with CFSE as described above and co-cultured with induced human Tregs. The cells were stimulated either with insulin mimetopes (100 ng ml^−1^) or the natural insulin B:9-23 epitope (10 μg ml^−1^).

Additional experiments were performed using effector T cells from T1D individuals and polyclonal stimulation as outlined above.

### Engraftment of NSG mice with human haematopoietic stem cells

Two-week-old NSG-HLA-DQ8 mice were reconstituted with at least 5 × 10^4^ CD34^+^HSCs from an *HLA-DQ8*^+^ donor per mouse by intravenous injection in 50 μl PBS into the retro orbital sinus without prior conditioning by irradiation or busulfan treatment. To avoid sex incompatibilities the sex of the NSG-HLA-DQ8 mice for reconstitution was chosen in accordance with the cord blood donor.

### Assessment of reconstitution efficacy in NSG-HLA-DQ8 mice

NSG-DQ8 mice were bled 5 and 8 weeks post engraftment and peripheral blood was analysed by FACS to characterize the engraftment of the human immune system using fluorescently labelled-specific human versus murine CD45 antibodies.

### Analyses of reconstituted humanized NSG-HLA-DQ8 mice

At various time points after reconstitution humanized NSG-HLA-DQ8 mice were euthanized and whole blood, peripheral lymph nodes, spleen and WAT were analysed for the presence of CD4^+^T cells. CD4^+^T cells were extracted from WAT by collagenase II (Sigma Aldrich, 4 mg ml^−1^) digestion and peripheral lymph nodes were homogenized by gentle grinding through a cell strainer followed by cellular FACS stainings and analyses as described above.

### Human *in vivo* Treg induction in humanized mice

Humanized NSG-HLA-DQ8 mice at 20 weeks post reconstitution were then subjected to *in vivo* Treg induction assays using insulin mimetope peptide infusion by subcutaneous implantation of osmotic mini-pumps, which permit the continuous delivery of minute amounts of peptide for 14 days 15,17. Mice were infused with a combination of ins.mim.1=14E-21G-22E and ins.mim.4=14E-21E-22E at 5 μg day^−1^. Control animals were infused with PBS. Successfully reconstituted animals were randomized to test groups for antigen-specific Treg induction. No animals were excluded due to illness or outlier results; therefore, no exclusion determination was required. For *ex vivo* T cell analyses, the entire group of mice treated with PBS or the insulin mimetopes was analysed. After 3 weeks, Foxp3^+^Treg induction was assessed upon insulin-specific tetramer stainings as described above and Tregs were identified based on CD4^+^CD3^+^CD127^low^CD25^+^. Treg identity was verified by intracellular staining for Foxp3 and by analyses of *Foxp3* mRNA abundance.

### Analysis of Treg signature genes

T cells were sort-purified; cDNA synthesis and subsequent amplification were performed using the SMARTer ultra-low input RNA Kit for sequencing—v3 (Takara Clontech) according to the manufactureŕs instructions. cDNA was purified using Agencourt AMPure XP Beads (Beckman Coulter). qPCR was performed on a CFX96 real time system (BioRad) using QuantiTect Primer assays (Qiagen) for *Foxp3, CTLA4, Il2-Rα, TIGIT, RTKN2, IKZF2, ENTPD1* and *FCRL3* and SsoFast Evagreen Supermix (BioRad). Levels of *Histone 3* and *18s* were used to normalize target gene expression levels (Histone: H3F3A BT020962, primers: fwd: 5′-ACTGGCTACAAAAGCCGCTC-3′; rev: 5′-ACTTGCCTCCTGCAAAGCAC-3′; 18 s: QuantiTect Primer assay, Qiagen).

### Analysis of T-cell effector genes

T cell effector genes were analysed on the same cDNA samples used for Treg signature gene analysis described above. qPCR was performed on a CFX96 real time system (BioRad) using QuantiTect Primer assays (Qiagen) for *IL17-Rα, NFATc2, IL-21, RORγt, T-bet* and *IFNγ* and SsoFast Evagreen Supermix (BioRad). Levels of *Histone 3* and *18s* were used to normalize target gene expression abundance (Histone: H3F3A BT020962, primers: fwd: 5′-ACTGGCTACAAAAGCCGCTC-3′, rev: 5′-ACTTGCCTCCTGCAAAGCAC-3′; 18 s: QuantiTect Primer assay, Qiagen).

### HLA fast genotyping

HLA-genotyping of the children was available. Fast genotyping was used for cord blood experiments and a protocol was developed on the basis of Nguyen *et al.*^70^. In brief, DNA was extracted from whole blood using the Quick-gDNA MiniPrep Kit (Zymo Research) according to the manufacturer's protocol. For qPCR analyses SsoAdvance Universal Probes Supermix (BioRad) was used with 15 ng of gDNA, 250 nM forward and reverse primer and 500 nM of Probes FAM and HEX. Standards were added for subsequent analysis with Bio-Rad CFX Manager 3.1. Primers: rs3104413 fwd 5′-CAGCTGAGCACTGAGTAG-3′, rs3104413 rev 5′-GCAGTTGAGAAGTGAGAG-3′, rs2854275 fwd 5′-CCAGAACCAAGCCTTAAC-3′, rs2854275 rev 5′-GCATCATCCTAGTGTCTAAC-3′, rs9273363 fwd 5′-GAGGGAGAAGGAAGATG-3′, rs9273363 rev 5′-GAAGCTGGTCTACATCTC-3′. Probes: FAM-Probe rs3104413 LPC [6FAM]CAGCCT[+G]CT[+C]TC[+C]TA[+T]TGG[BHQ1], HEX-Probe rs3104413 LPG [HEX]CAGCCT[+G]CT[+G]TC[+C]TA[+T]TGG[BHQ1], FAM-Probe rs2854275 G [6FAM]TCCACA[+T]TT[+C]AC[+A]AG[+A]AGA[BHQ1], HEX-Probe rs2854275 T [HEX]TCCACA[+T]TT[+A]AC[+A]AG[+A]AGA[BHQ1], FAM-Probe rs9273363 LPA [6FAM]CATGGC[+C]TT[+A]CA[+T]AA[+C]CTC[BHQ1], HEX-Probe rs9273363 LPC [HEX]CATGGC[+C]TT[+C]CA[+T]AA[+C]CTC[BHQ1].

### DNA bisulfite conversion and methylation analysis

Because of the reduced nature of available sample material, FACS-sorted CD4^+^T cells (50–2,000 cells) were subjected to a combined sample lysis and bisulfite conversion using the EpiTect Plus LyseAll Bisulfite Kit (Qiagen, Hilden, Germany) or the EZ DNA Methylation-Direct Kit (Zymo Research) according to the manufacturer's instructions. For bias-controlled quantitative methylation analysis, a combination of MS-HRM and subsequent Pyrosequencing was performed as described earlier^42^,^43^. Utilizing the PyroMark Assay Design Software 2.0 (Qiagen), PCR primers (human forward: 5′-AAGTTGAATGGGGGATGTTTTTGGGATATAGATTATG-3′; human reverse: 5′-CTACCACATCCACCAACACCCATATCACC-3′; annealing-temperature: 62 °C; murine forward: 5′-TTGGGTTTTGTTGTTATAATTTGAATTTGG-3′; murine reverse: 5′-ACCTACCTAATACTCACCAAACATC-3′; annealing-temperature: 60 °C) and the according sequencing primer (human: 5′-TAGTTTTAGATTTGTTTAGATTTT-3′; murine: 5′-AATTTGAATTTGGTTAGATTTTT-3′) were designed to cover the area of differential methylation in the first *Foxp3* intron initially reported by Baron *et al.*^42^ (Supplementary Fig. 14). Pyrosequencing data are presented as means of all CpG-sites analysed due to high homology between methylation levels of the individual sites (Supplementary Fig. 14).

### Statistics

Results are presented as mean and s.e.m. or as percentages, where appropriate. For normally distributed data, Student's *t*-test for unpaired values was used to compare means between independent groups and the Student's *t*-test for paired values was used to compare values for the same sample or subject tested under different conditions. The non-parametric Wilcoxon signed-ranks test was applied when data did not show Gaussian distribution. Group size estimations were based upon a power calculation to minimally yield an 80% chance to detect a significant difference in the respective parameter of *P*<0.05 between the relevant groups. For all tests, a two-tailed *P* value of <0.05 was considered to be significant. Statistical significance is shown as *=*P*<0.05; **=*P*<0.01; ***=*P*<0.001, or not significant (ns) *P*> 0.05. Analyses were performed using the programs GraphPad Prism 6 (La Jolla, CA) and the Statistical Package for the Social Sciences (SPSS 19.0; SPSS Inc., Chicago, IL).

## Additional information

**How to cite this article:** Serr, I. *et al.* Type 1 diabetes vaccine candidates promote human Foxp3^+^Treg induction in humanized mice. *Nat. Commun.* 7:10991 doi: 10.1038/ncomms10991 (2016).

## Supplementary Material

Supplementary Information Supplementary Figures 1-18

## References

[b1] Bluestone J. A., Herold K. & Eisenbarth G. Genetics, pathogenesis and clinical interventions in type 1 diabetes. Nature 464, 1293–1300 (2010) . 20432533 10.1038/nature08933 PMC4959889

[b2] Patterson C. C. *et al.* Incidence trends for childhood type 1 diabetes in Europe during 1989-2003 and predicted new cases 2005-20: a multicentre prospective registration study. Lancet 373, 2027–2033 (2009) . 19481249 10.1016/S0140-6736(09)60568-7

[b3] Fairchild P. J., Wildgoose R., Atherton E., Webb S. & Wraith D. C. An autoantigenic T cell epitope forms unstable complexes with class II MHC: a novel route for escape from tolerance induction. Int. Immunol. 5, 1151–1158 (1993) . 7694643 10.1093/intimm/5.9.1151

[b4] Garcia K. C., Teyton L. & Wilson I. A. Structural basis of T cell recognition. Annu. Rev. Immunol. 17, 369–397 (1999) . 10358763 10.1146/annurev.immunol.17.1.369

[b5] Hahn M., Nicholson M. J., Pyrdol J. & Wucherpfennig K. W. Unconventional topology of self peptide-major histocompatibility complex binding by a human autoimmune T cell receptor. Nat. Immunol. 6, 490–496 (2005) . 15821740 10.1038/ni1187 PMC3415330

[b6] Liu G. Y. *et al.* Low avidity recognition of self-antigen by T cells permits escape from central tolerance. Immunity 3, 407–415 (1995) . 7584132 10.1016/1074-7613(95)90170-1

[b7] Stadinski B. D. *et al.* Diabetogenic T cells recognize insulin bound to IAg7 in an unexpected, weakly binding register. Proc. Natl Acad. Sci. USA 107, 10978–10983 (2010) . 20534455 10.1073/pnas.1006545107 PMC2890771

[b8] Ziegler A. G. *et al.* Seroconversion to multiple islet autoantibodies and risk of progression to diabetes in children. JAMA 309, 2473–2479 (2013) . 23780460 10.1001/jama.2013.6285 PMC4878912

[b9] Ziegler A. G. & Nepom G. T. Prediction and pathogenesis in type 1 diabetes. Immunity 32, 468–478 (2010) . 20412757 10.1016/j.immuni.2010.03.018 PMC2861716

[b10] Fontenot J. D., Gavin M. A. & Rudensky A. Y. Foxp3 programs the development and function of CD4^+^CD25^+^ regulatory T cells. Nat. Immunol. 4, 330–336 (2003) . 12612578 10.1038/ni904

[b11] Khattri R., Cox T., Yasayko S. A. & Ramsdell F. An essential role for Scurfin in CD4^+^CD25^+^ T regulatory cells. Nat. Immunol. 4, 337–342 (2003) . 12612581 10.1038/ni909

[b12] Roncador G. *et al.* Analysis of FOXP3 protein expression in human CD4^+^CD25^+^ regulatory T cells at the single-cell level. Eur. J. Immunol. 35, 1681–1691 (2005) . 15902688 10.1002/eji.200526189

[b13] Levings M. K., Sangregorio R. & Roncarolo M. G. Human cd25(+)cd4(+) t regulatory cells suppress naive and memory T cell proliferation and can be expanded *in vitro* without loss of function. J. Exp. Med. 193, 1295–1302 (2001) . 11390436 10.1084/jem.193.11.1295 PMC2193376

[b14] Modigliani Y. *et al.* Lymphocytes selected in allogeneic thymic epithelium mediate dominant tolerance toward tissue grafts of the thymic epithelium haplotype. Proc. Natl Acad. Sci. USA 92, 7555–7559 (1995) . 7638230 10.1073/pnas.92.16.7555 PMC41378

[b15] Daniel C. & von Boehmer H. Extra-thymically induced regulatory T cells: do they have potential in disease prevention? Semin. Immunol. 23, 410–417 (2011) . 21724411 10.1016/j.smim.2011.06.004 PMC3230715

[b16] Daniel C. & von Boehmer H. Extrathymic generation of regulatory T cells--chances and challenges for prevention of autoimmune disease. Adv. Immunol. 112, 177–213 (2011) . 22118409 10.1016/B978-0-12-387827-4.00005-X

[b17] Daniel C., Weigmann B., Bronson R. & von Boehmer H. Prevention of type 1 diabetes in mice by tolerogenic vaccination with a strong agonist insulin mimetope. J. Exp. Med. 208, 1501–1510 (2011) . 21690251 10.1084/jem.20110574 PMC3135372

[b18] von Boehmer H. & Daniel C. Therapeutic opportunities for manipulating T(Reg) cells in autoimmunity and cancer. Nat. Rev. Drug Discov. 12, 51–63 (2013) . 23274471 10.1038/nrd3683

[b19] Kretschmer K. *et al.* Inducing and expanding regulatory T cell populations by foreign antigen. Nat. Immunol. 6, 1219–1227 (2005) . 16244650 10.1038/ni1265

[b20] Daniel C., Ploegh H. & von Boehmer H. Antigen-specific induction of regulatory T cells *in vivo* and *in vitro*. Methods Mol. Biol. 707, 173–185 (2011) . 21287335 10.1007/978-1-61737-979-6_11

[b21] Daniel C., Wennhold K., Kim H. J. & von Boehmer H. Enhancement of antigen-specific Treg vaccination *in vivo*. Proc. Natl Acad. Sci. USA 107, 16246–16251 (2010) . 20805478 10.1073/pnas.1007422107 PMC2941325

[b22] Gottschalk R. A., Corse E. & Allison J. P. TCR ligand density and affinity determine peripheral induction of Foxp3 *in vivo*. J. Exp. Med. 207, 1701–1711 (2010) . 20660617 10.1084/jem.20091999 PMC2916126

[b23] Nakayama M. *et al.* Prime role for an insulin epitope in the development of type 1 diabetes in NOD mice. Nature 435, 220–223 (2005) . 15889095 10.1038/nature03523 PMC1364531

[b24] Jaeckel E., Lipes M. A. & von Boehmer H. Recessive tolerance to preproinsulin 2 reduces but does not abolish type 1 diabetes. Nat. Immunol. 5, 1028–1035 (2004) . 15378058 10.1038/ni1120

[b25] Alleva D. G. *et al.* A disease-associated cellular immune response in type 1 diabetics to an immunodominant epitope of insulin. J. Clin. Invest. 107, 173–180 (2001) . 11160133 10.1172/JCI8525 PMC198872

[b26] Daniel D., Gill R. G., Schloot N. & Wegmann D. Epitope specificity, cytokine production profile and diabetogenic activity of insulin-specific T cell clones isolated from NOD mice. Eur. J. Immunol. 25, 1056–1062 (1995) . 7537670 10.1002/eji.1830250430

[b27] Wegmann D. R., Norbury-Glaser M. & Daniel D. Insulin-specific T cells are a predominant component of islet infiltrates in pre-diabetic NOD mice. Eur. J. Immunol. 24, 1853–1857 (1994) . 8056042 10.1002/eji.1830240820

[b28] Crawford F. *et al.* Specificity and detection of insulin-reactive CD4^+^ T cells in type 1 diabetes in the nonobese diabetic (NOD) mouse. Proc. Natl Acad. Sci. USA 108, 16729–16734 (2011) . 21949373 10.1073/pnas.1113954108 PMC3189014

[b29] Lee K. H., Wucherpfennig K. W. & Wiley D. C. Structure of a human insulin peptide-HLA-DQ8 complex and susceptibility to type 1 diabetes. Nat. Immunol. 2, 501–507 (2001) . 11376336 10.1038/88694

[b30] Yang J. *et al.* Autoreactive T cells specific for insulin B:11-23 recognize a low-affinity peptide register in human subjects with autoimmune diabetes. Proc. Natl Acad. Sci. USA 111, 14840–14845 (2014) . 25267644 10.1073/pnas.1416864111 PMC4205657

[b31] Nakayama M. *et al.* Regulatory versus inflammatory cytokine T-cell responses to mutated insulin peptides in healthy and type 1 diabetic subjects. Proc. Natl Acad. Sci. USA 112, 4429–4434 (2015) . 25831495 10.1073/pnas.1502967112 PMC4394309

[b32] Durinovic-Bello I. *et al.* DRB1*0401-restricted human T cell clone specific for the major proinsulin73-90 epitope expresses a down-regulatory T helper 2 phenotype. Proc. Natl Acad. Sci. USA 103, 11683–11688 (2006) . 16868084 10.1073/pnas.0603682103 PMC1544230

[b33] Mannering S. I. *et al.* Current approaches to measuring human islet-antigen specific T cell function in type 1 diabetes. Clin. Exp. Immunol. 162, 197–209 (2010) . 20846160 10.1111/j.1365-2249.2010.04237.x PMC2996587

[b34] Maus M. V., Riley J. L., Kwok W. W., Nepom G. T. & June C. H. HLA tetramer-based artificial antigen-presenting cells for stimulation of CD4^+^ T cells. Clin. Immunol. 106, 16–22 (2003) . 12584046 10.1016/s1521-6616(02)00017-7

[b35] Sauer S. *et al.* T cell receptor signaling controls Foxp3 expression via PI3K, Akt, and mTOR. Proc. Natl Acad. Sci. USA 105, 7797–7802 (2008) . 18509048 10.1073/pnas.0800928105 PMC2409380

[b36] Pearson T., Greiner D. L. & Shultz L. D. in *Current Protocols* *in Immunology* (eds Coligan, J. E. *et al.*) Ch. 15, Unit 15, 21 (Wiley, 2008) .

[b37] Ferraro A. *et al.* Interindividual variation in human T regulatory cells. Proc. Natl Acad. Sci. USA 111, E1111–E1120 (2014) . 24610777 10.1073/pnas.1401343111 PMC3970507

[b38] Pfoertner S. *et al.* Signatures of human regulatory T cells: an encounter with old friends and new players. Genome Biol. 7, R54 (2006) . 16836768 10.1186/gb-2006-7-7-r54 PMC1779567

[b39] Yu X. *et al.* The surface protein TIGIT suppresses T cell activation by promoting the generation of mature immunoregulatory dendritic cells. Nat. Immunol. 10, 48–57 (2009) . 19011627 10.1038/ni.1674

[b40] Stanietsky N. *et al.* The interaction of TIGIT with PVR and PVRL2 inhibits human NK cell cytotoxicity. Proc. Natl Acad. Sci. USA 106, 17858–17863 (2009) . 19815499 10.1073/pnas.0903474106 PMC2764881

[b41] Deaglio S. *et al.* Adenosine generation catalyzed by CD39 and CD73 expressed on regulatory T cells mediates immune suppression. J. Exp. Med. 204, 1257–1265 (2007) . 17502665 10.1084/jem.20062512 PMC2118603

[b42] Baron U. *et al.* DNA demethylation in the human FOXP3 locus discriminates regulatory T cells from activated FOXP3(+) conventional T cells. Eur. J. Immunol. 37, 2378–2389 (2007) . 17694575 10.1002/eji.200737594

[b43] Floess S. *et al.* Epigenetic control of the foxp3 locus in regulatory T cells. PLoS Biol. 5, e38 (2007) . 17298177 10.1371/journal.pbio.0050038 PMC1783672

[b44] Collison L. W. & Vignali D. A. *In vitro* Treg suppression assays. Methods Mol. Biol. 707, 21–37 (2011) . 21287326 10.1007/978-1-61737-979-6_2 PMC3043080

[b45] Skyler J. S. *et al.* Effects of oral insulin in relatives of patients with type 1 diabetes: the Diabetes Prevention Trial--Type 1. Diabetes Care 28, 1068–1076 (2005) . 15855569 10.2337/diacare.28.5.1068

[b46] Harrison L. C. *et al.* Pancreatic beta-cell function and immune responses to insulin after administration of intranasal insulin to humans at risk for type 1 diabetes. Diabetes care 27, 2348–2355 (2004) . 15451899 10.2337/diacare.27.10.2348

[b47] Nanto-Salonen K. *et al.* Nasal insulin to prevent type 1 diabetes in children with HLA genotypes and autoantibodies conferring increased risk of disease: a double-blind, randomised controlled trial. Lancet 372, 1746–1755 (2008) . 18814906 10.1016/S0140-6736(08)61309-4

[b48] Diabetes Prevention Trial--Type 1 Diabetes Study Group. Effects of insulin in relatives of patients with type 1 diabetes mellitus. N. Engl. J. Med. 346, 1685–1691 (2002) . 12037147 10.1056/NEJMoa012350

[b49] Bonifacio E. *et al.* Effects of high-dose oral insulin on immune responses in children at high risk for type 1 diabetes: the Pre-POINT randomized clinical trial. JAMA. 313, 1541–1549 (2015) . 25898052 10.1001/jama.2015.2928

[b50] Shiina T., Inoko H. & Kulski J. K. An update of the HLA genomic region, locus information and disease associations: 2004. Tissue Antigens 64, 631–649 (2004) . 15546336 10.1111/j.1399-0039.2004.00327.x

[b51] Achenbach P. *et al.* Characteristics of rapid vs slow progression to type 1 diabetes in multiple islet autoantibody-positive children. Diabetologia 56, 1615–1622 (2013) . 23539116 10.1007/s00125-013-2896-y

[b52] Thrower S. L. *et al.* Proinsulin peptide immunotherapy in type 1 diabetes: report of a first-in-man phase I safety study. Clin. Exp. Immunol. 155, 156–165 (2009) . 19040615 10.1111/j.1365-2249.2008.03814.x PMC2675245

[b53] Gottschalk R. A. *et al.* Distinct influences of peptide-MHC quality and quantity on *in vivo* T-cell responses. Proc. Natl Acad. Sci. USA 109, 881–886 (2012) . 22223661 10.1073/pnas.1119763109 PMC3271915

[b54] Singh N. J. & Schwartz R. H. The strength of persistent antigenic stimulation modulates adaptive tolerance in peripheral CD4^+^ T cells. J. Exp. Med. 198, 1107–1117 (2003) . 14530379 10.1084/jem.20030913 PMC2194218

[b55] Stefanova I. *et al.* TCR ligand discrimination is enforced by competing ERK positive and SHP-1 negative feedback pathways. Nat. Immunol. 4, 248–254 (2003) . 12577055 10.1038/ni895

[b56] Sullivan S. P. *et al.* Dissolving polymer microneedle patches for influenza vaccination. Nat. Med. 16, 915–920 (2010) . 20639891 10.1038/nm.2182 PMC2917494

[b57] Gupta J., Felner E. I. & Prausnitz M. R. Rapid pharmacokinetics of intradermal insulin administered using microneedles in type 1 diabetes subjects. Diabetes Technol. Ther. 13, 451–456 (2011) . 21355717 10.1089/dia.2010.0204 PMC3131988

[b58] Hirobe S. *et al.* Development and clinical study of a self-dissolving microneedle patch for transcutaneous immunization device. Pharm. Res. 30, 2664–2674 (2013) . 23775442 10.1007/s11095-013-1092-6

[b59] Norman J. J. *et al.* Microneedle patches: usability and acceptability for self-vaccination against influenza. Vaccine 32, 1856–1862 (2014) . 24530146 10.1016/j.vaccine.2014.01.076 PMC3979961

[b60] Marodon G. *et al.* High diversity of the immune repertoire in humanized NOD.SCID.gamma c−/− mice. Eur. J. Immunol. 39, 2136–2145 (2009) . 19572320 10.1002/eji.200939480

[b61] Shultz L. D., Ishikawa F. & Greiner D. L. Humanized mice in translational biomedical research. Nat. Rev. Immunol. 7, 118–130 (2007) . 17259968 10.1038/nri2017

[b62] Ziegler A. G., Bonifacio E. & BABYDIAB- BABYDIET Study Group. Age-related islet autoantibody incidence in offspring of patients with type 1 diabetes. Diabetologia 55, 1937–1943 (2012) . 22289814 10.1007/s00125-012-2472-x

[b63] Ziegler A. G., Hummel M., Schenker M. & Bonifacio E. Autoantibody appearance and risk for development of childhood diabetes in offspring of parents with type 1 diabetes: the 2-year analysis of the German BABYDIAB Study. Diabetes 48, 460–468 (1999) . 10078544 10.2337/diabetes.48.3.460

[b64] Achenbach P. *et al.* Autoantibodies to zinc transporter 8 and SLC30A8 genotype stratify type 1 diabetes risk. Diabetologia 52, 1881–1888 (2009) . 19590848 10.1007/s00125-009-1438-0

[b65] Nabozny G. H. *et al.* HLA-DQ8 transgenic mice are highly susceptible to collagen-induced arthritis: a novel model for human polyarthritis. J. Exp. Med. 183, 27–37 (1996) . 8551230 10.1084/jem.183.1.27 PMC2192409

[b66] Covassin L. *et al.* Human immune system development and survival of non-obese diabetic (NOD)-scid IL2rgamma(null) (NSG) mice engrafted with human thymus and autologous haematopoietic stem cells. Clin. Exp. Immunol. 174, 372–388 (2013) . 23869841 10.1111/cei.12180 PMC3826304

[b67] Ettinger R. A. & Kwok W. W. A peptide binding motif for HLA-DQA1*0102/DQB1*0602, the class II MHC molecule associated with dominant protection in insulin-dependent diabetes mellitus. J. Immunol. 160, 2365–2373 (1998) . 9498778

[b68] Sidney J. *et al.* in *Current* *Protocols in Immunology* (eds Coligan, J. E. *et al.*) Ch. 18, Unit 18, 13 (Wiley, 2013) .

[b69] Day C. L. *et al.* *Ex vivo* analysis of human memory CD4 T cells specific for hepatitis C virus using MHC class II tetramers. J. Clin. Invest. 112, 831–842 (2003) . 12975468 10.1172/JCI18509 PMC193667

[b70] Nguyen C., Varney M. D., Harrison L. C. & Morahan G. Definition of high-risk type 1 diabetes HLA-DR and HLA-DQ types using only three single nucleotide polymorphisms. Diabetes 62, 2135–2140 (2013) . 23378606 10.2337/db12-1398 PMC3661605

