# Supplementary material for: Type 1 diabetes vaccine candidates promote human Foxp3+Treg induction in humanized mice
Source: Nat Commun. 2016 Mar 15;7:10991. doi: 10.1038/ncomms10991 (PMC4796321; doi:10.1038/ncomms10991)
Supplement: Supplementary Information — Supplementary Figures 1-18 [file ncomms10991-s1.pdf]

Supplementary Figures

Suppl. Fig.1

**ins.mim.1 (14E-21G-22E):**	HLVE**E**LYLVCG**GE**G
**ins.mim.2 (21G-22E):**	HLVEALYLVCG**GE**G
**ins.mim.3 (21E-22E):**	HLVEALYLVCGE**E**G
**ins.mim.4 (14E-21E-22E):**	HLVE**E**LYLVCGE**E**G
**insulin B chain epitope:**	HLVEALYLVCGERG

Supplementary Figure 1. Shown are peptide sequences of insulin B chain 10-23 mimetopes using one letter amino acid codes.

Suppl. Fig.2

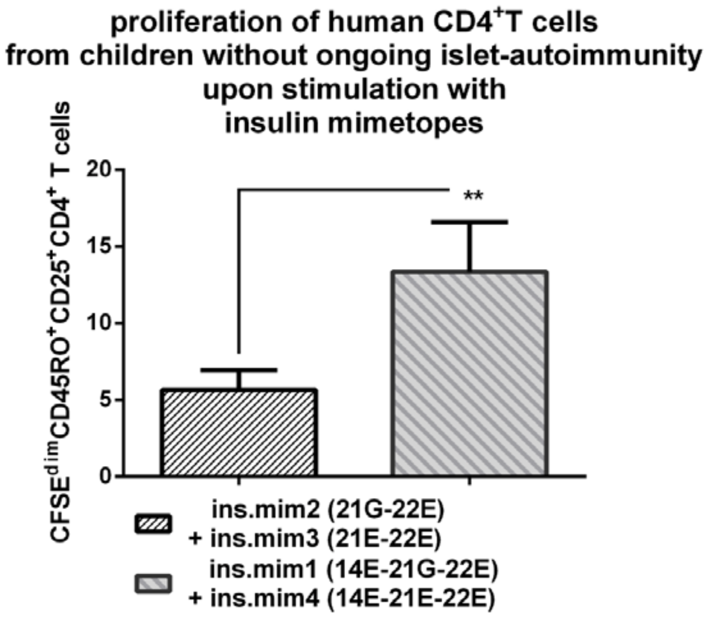

Supplementary Figure 2. Proliferation of human CD4$^+$T cells from children without ongoing islet autoimmunity upon stimulation with insulin mimetopes. Percentages of divided human CFSE^{dim}CD4$^+$CD45RO$^+$T cells upon stimulation with a combination of ins.mim.2= 21G-22E and ins.mim.3=21E-22E or a combination of ins.mim.1= 14E-21G-22E and ins.mim.4=14E-21E-22E. Bars represent the means ± s.e.m. (n=5) from duplicate wells of five children and four independent experiments. ** $P < 0.01$ (Student´s *t*-test).

Suppl.Fig.3

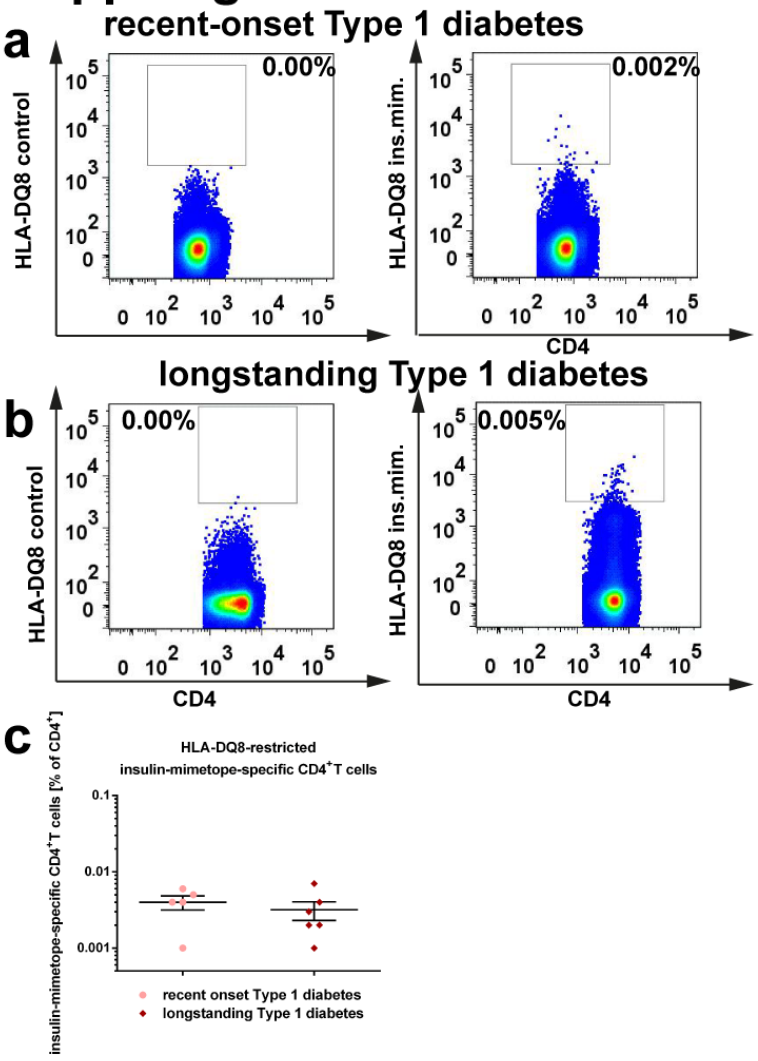

Supplementary Figure 3. Representative FACS plots of HLA-DQ8-restricted insulin mimetope-specific CD4^{+}T cells in a child with recent onset of Type 1 diabetes (a) or longterm Type 1 diabetes (b) using tetramer and respective control stainings. (c) Frequencies of HLA-DQ8-restricted insulin mimetope-specific CD4^{+}T cells from children with recent onset vs. longstanding Type 1 diabetes. FACS analyses were performed using samples from five children with recent onset of Type 1 diabetes and six children with longterm diabetes in five independent experiments (mean±s.e.m).

Suppl.Fig.4

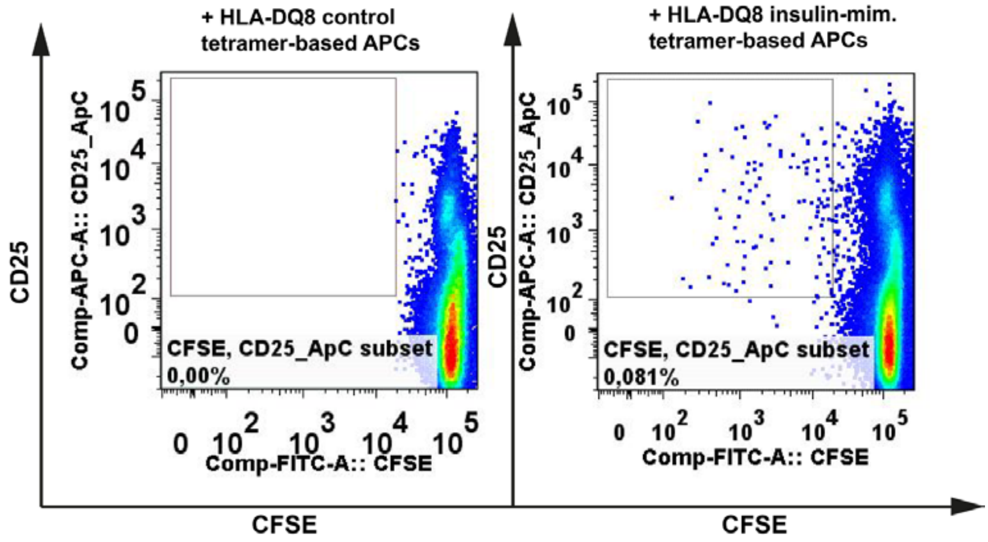

Supplementary Figure 4. Proliferative responses of CD4$^+$T cells from children without ongoing islet autoimmunity to HLA-DQ8-restricted insulin mimetope-specific artificial antigen-presenting cells. Representative FACS plots for CFSE dilution profiles of human CD4$^+$T cells from children without autoimmunity (islet autoantibody negative) stimulated with HLA-DQ8 control (left) or HLA-DQ8 insulin mimetope-specific (right) artificial antigen-presenting cells. Responsive CD4$^+$T cells were defined as proliferating CFSE^{dim}CD4$^+$CD25$^+$CD45RO$^+$T cells.

Suppl.Fig.5

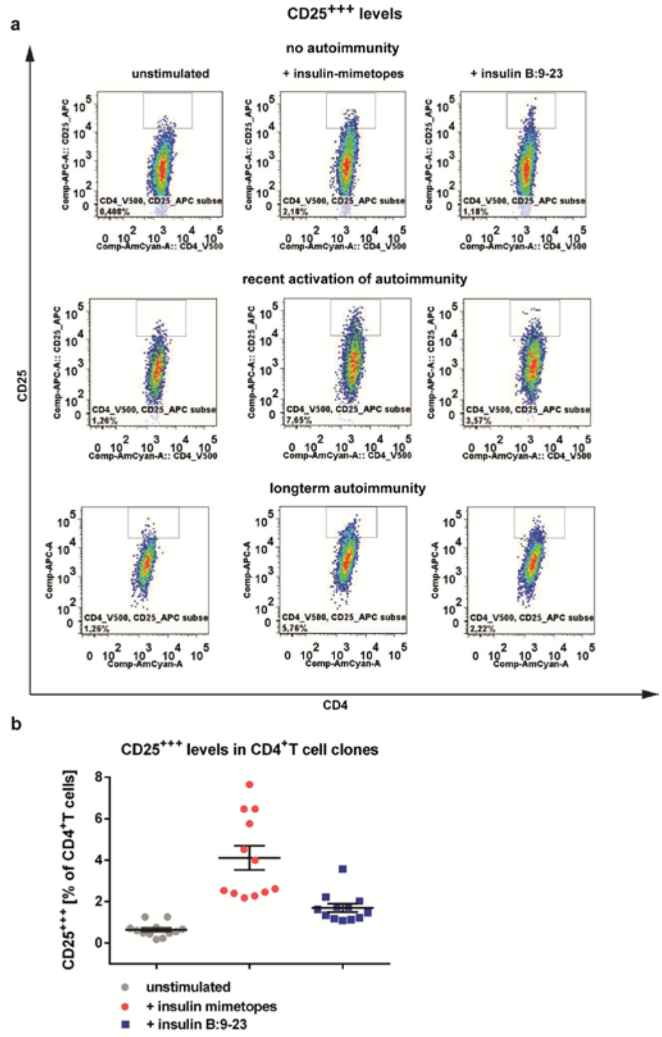

Supplementary Figure 5. Agonistic activity of insulin mimetopes in human insulin-specific CD4$^+$T cell clones compared to insulin B:9-23. a) CD25^{+++} levels of insulin-specific CD4$^+$T cell clones either left unstimulated (control, left plot) or upon stimulation with insulin mimetopes (ins.mim.1, 2, 3,4 at final 100 ng per ml, middle plot) or with insulin B:9-23 (1000 ng per ml, right plot). Insulin-specific CD4$^+$T cell clones from children without ongoing autoimmunity, with recent activation or with longterm autoimmunity. **b)** Summary graph for CD25^{+++} levels (mean±s.e.m). Triplicate wells have been analyzed per condition. Four clones from two children without ongoing autoimmunity, four clones from two children with recent activation and four clones from four children with longterm autoimmunity. Four independent experiments have been performed.

Suppl.Fig. 6

Sorting strategy for human naive CD4^{+}T-cells

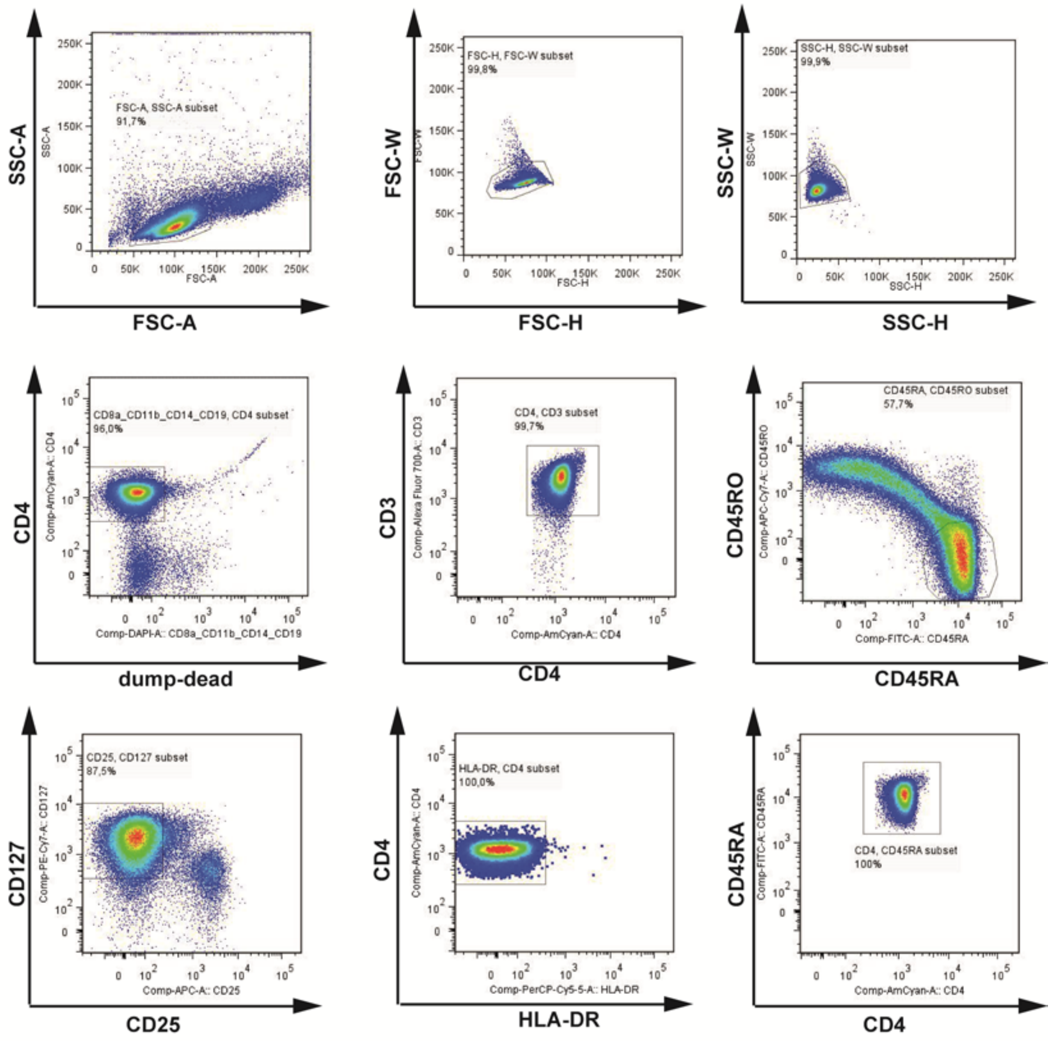

Supplementary Figure 6. Gating strategy for the sort purification of human naïve CD4^{+}T cells used in *in vitro* Treg induction assays. Human naïve CD4^{+}T cells were sorted to 99.9% purity as CD4^{+}CD3^{+}CD45RA^{+}CD45RO^{-}CD127^{+}CD25^{-}HLA-DR^{-} T cells using a BD FACSARIA III cell sorting system.

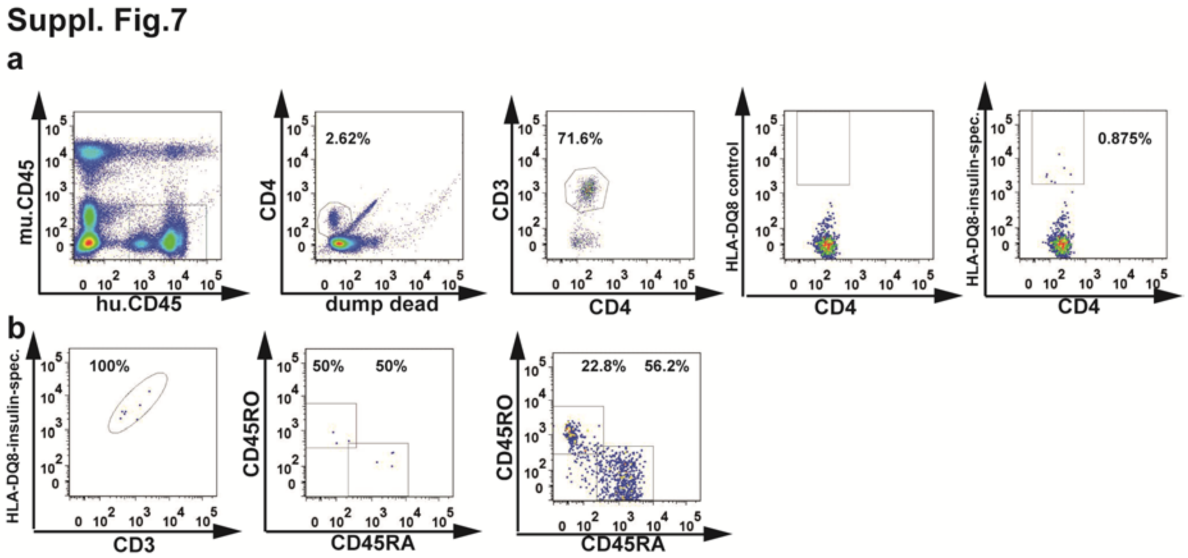

Supplementary Figure 7. Characterization of HLA-DQ8-restricted insulin mimetope-specific CD4$^+$T cell responses in NSG-HLA-DQ8 mice. Human immune subsets purified from peripheral blood (**a**+**b**) of humanized NSG-HLA-DQ8 mice, 20 weeks post reconstitution, are first identified based on murine vs. human CD45 expression. Human CD4$^+$CD3$^+$T cells were characterized upon exclusion of dead cells and additional markers (CD8, CD11b, CD14, CD19). (**a**) Representative set of FACS plots for the identification of HLA-DQ8-restricted insulin-specific CD4$^+$T cells. (**b**) Representative set of FACS plots for the phenotypic characterization of blood-derived HLA-DQ8-restricted insulin-specific CD4$^+$T cells based on gating against CD3 and CD45RA vs. CD45RO expression (memory-status, insulin-specific vs. polyclonal).

Suppl.Fig.8

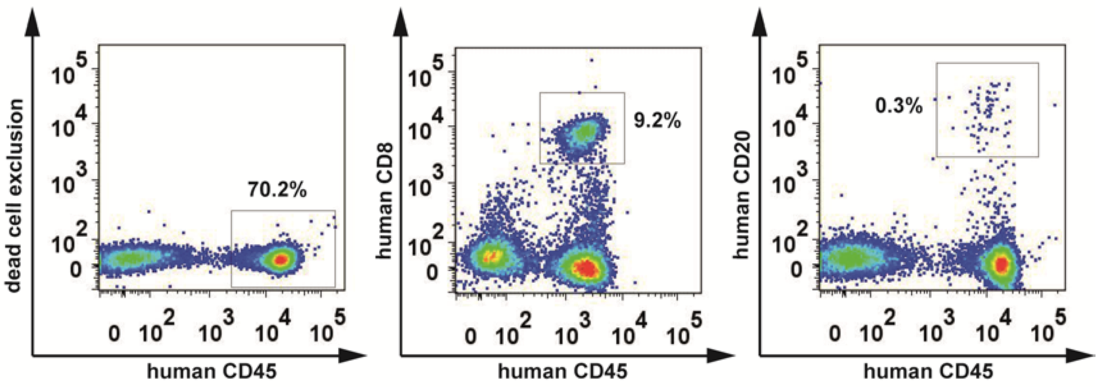

Supplementary Figure 8. Identification of immune subsets in reconstituted NSG-HLA-DQ8 mice. Human immune subsets purified from peripheral blood of NSG-HLA-DQ8 mice, 20 weeks post reconstitution, are first identified based on murine vs. human CD45 expression. Representative set of FACS plots for the identification of human $CD8^{+}$T cells and $CD20^{+}$B cells are shown.

Suppl. Fig.9

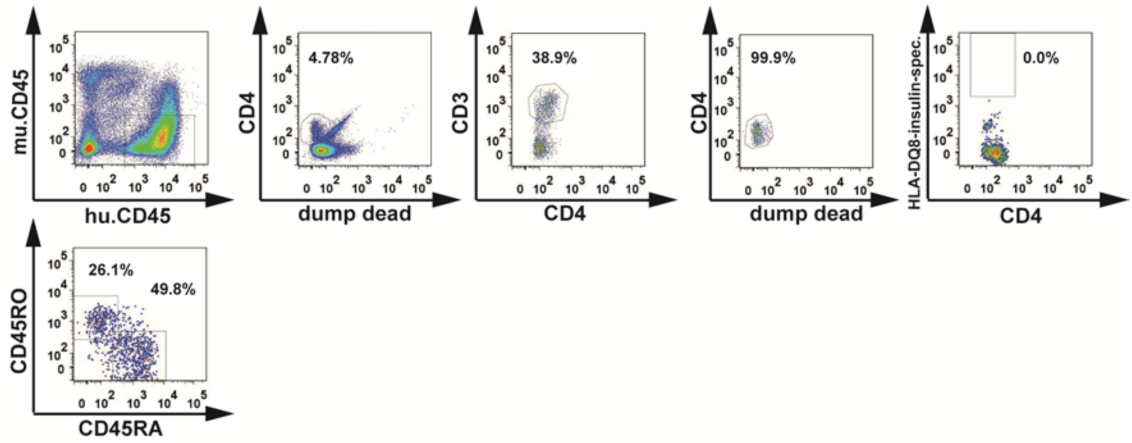

Supplementary Figure 9. Characterization of HLA-DQ8-restricted insulin mimetope-specific CD4$^+$T cell responses in NSG-HLA-DQ8 mice. Human immune subsets purified from visceral white fat tissues (**a+b**). (**a**) Representative set of FACS plots for the identification of HLA-DQ8-restricted insulin mimetope-specific CD4$^+$T cells purified from visceral white fat tissues. (**b**) Characterization of memory status (CD45RA vs. CD45RO expression (in polyclonal fat-derived CD4$^+$T cells).

Suppl.Fig.10

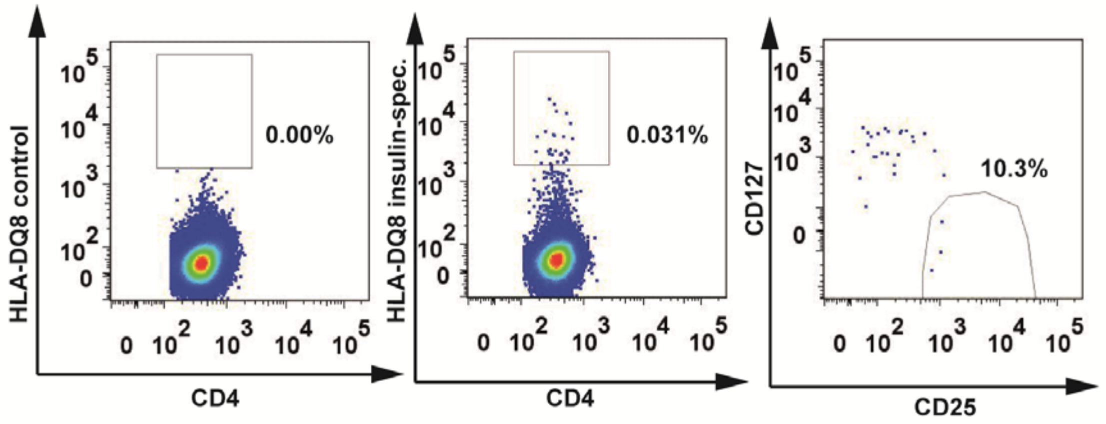

Supplementary Figure 10. Characterization of HLA-DQ8-restricted insulin mimetope-specific CD4$^+$T cell responses in NSG-HLA-DQ8 mice upon application of insulin mimetopes to humanized NSG-HLA-DQ8 mice for Treg induction *in vivo*. Treg induction in humanized mice was performed at 20 weeks post reconstitution. Three weeks after Treg induction using insulin mimetopes infusion by osmotic mini-pumps (ins.mim.1= 14E-21G-22E and ins.mim.4=14E-21E-22E) human immune subsets were purified from pancreatic and mesenteric lymph nodes of respective NSG mice. Human CD4$^+$CD3$^+$T cells were characterized upon exclusion of dead cells and additional markers (CD8, CD11b, CD14, CD19). Representative set of FACS plots for the identification of control- (left plot) and HLA-DQ8-restricted insulin mimetope-specific CD4$^+$CD127^{low}CD25^{high}Tregs are shown (total insulin mimetope-specific CD4$^+$T cells shown in middle plot, frequencies of CD127^{low}CD25^{high}Tregs in right plot).

Suppl.Fig.11

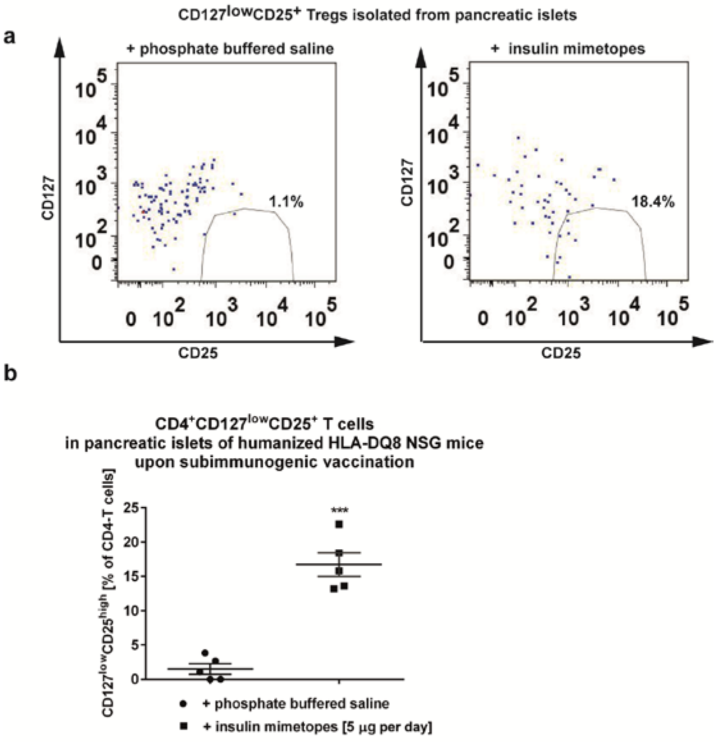

Supplementary Figure 11. Identification of human CD4^{+}T cells in pancreatic islets of humanized NSG-HLA-DQ8 mice. Human CD4^{+}T cells isolated from pancreatic islets of humanized NSG-HLA-DQ8 mice, 23 weeks post reconstitution were first identified flow cytometrically based on human CD45 expression. Human CD4^{+}CD3^{+}T cells were characterized upon exclusion of dead cells and additional markers (CD8, CD11b, CD14, CD19). (**a**) Representative set of FACS plots for the identification of pancreatic islet-infiltrating CD4^{+}CD3^{+}CD127^{low}CD25^{+}Tregs isolated from humanized NSG-HLA-DQ8 mice upon subimmunogenic Treg induction *in vivo* using insulin mimetopes infusion by osmotic mini-pumps (ins.mim1= 14E-21G-22E and ins.mim4=14E-21E-22E) or control (phosphate buffered saline). (**b**) Summary graph for the frequencies of infiltrating CD4^{+}CD3^{+}CD127^{low}CD25^{+}Tregs upon Treg induction using insulin mimetopes or control (phosphate buffered saline), mean±s.e.m.. n= 5 mice per group from two independent experiments; *** P<0.001 (Student´s *t*-test).

Suppl.Fig.12

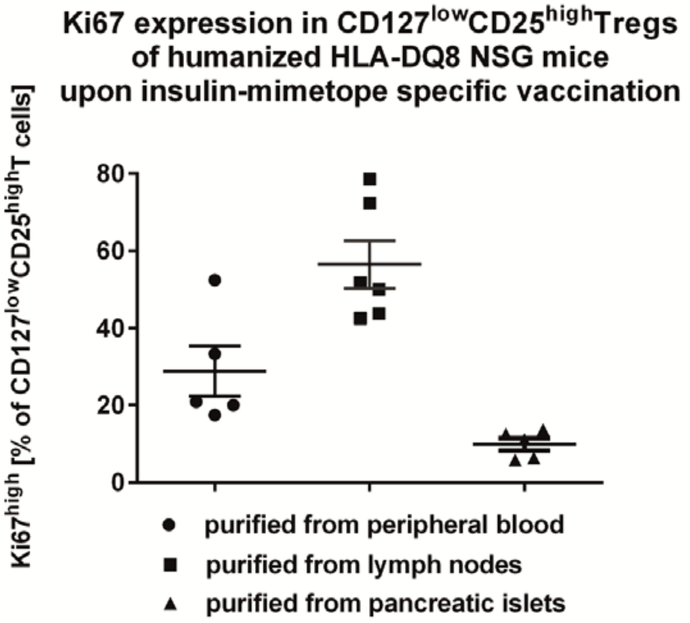

Supplementary Figure 12. Analysis of Ki67 expression levels in human CD127^{low}CD25^{high} Tregs. Treg induction in humanized mice was performed at 20 weeks post reconstitution. Three weeks after Treg induction using insulin mimetopes infusion by osmotic mini-pumps (ins.mim.1= 14E-21G-22E and ins.mim.4=14E-21E-22E) human immune subsets were purified from peripheral blood, pancreatic and mesenteric lymph nodes or from pancreatic islets of respective NSG mice. Human CD4^{+}CD3^{+}T cells were first characterized upon exclusion of dead cells and additional markers (CD8, CD11b, CD14, CD19). Summary graphs are shown for the analysis of the proliferative capacity of CD127^{low}CD25^{high}Treg cells by intracellular staining for Ki67 (mean±s.e.m.). n= 8 mice, from two independent experiments.

Suppl. Fig.13

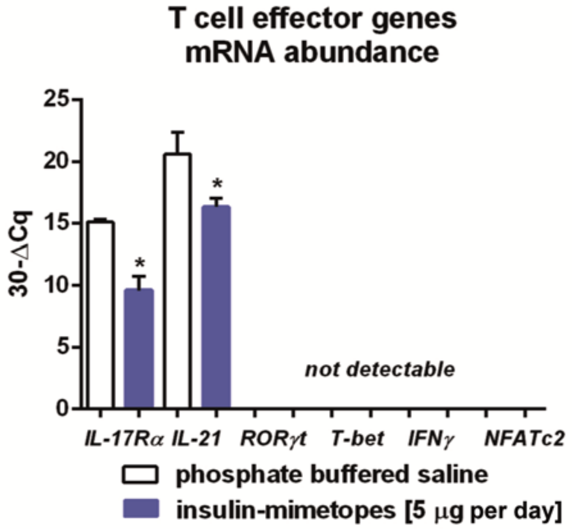

Supplementary Figure 13. Analysis of human T cell effector genes upon subimmunogenic vaccination with human insulin mimetopes in NSG-HLA-DQ8 transgenic mice. Quantitative RT-PCR analyses of *IL-17Rα, IL-21, RORγt, T-bet, IFN-γ* and *NFATc2* abundance in human $CD4^+$T cells purified from pooled spleens and lymph nodes of humanized mice after three weeks of *in vivo* Treg induction using subcutaneous insulin mimetopes infusion by osmotic mini-pumps (ins.mim.1= 14E-21G-22E and ins.mim.4=14E-21E-22E) in humanized NSG-HLA-DQ8 transgenic mice (n=4). Bars represent the means ± s.e.m. (n=4 mice per group and experiment, n=2 independent experiments). * $P<0.05$ (Student´s *t*-test).

Suppl.Fig.14

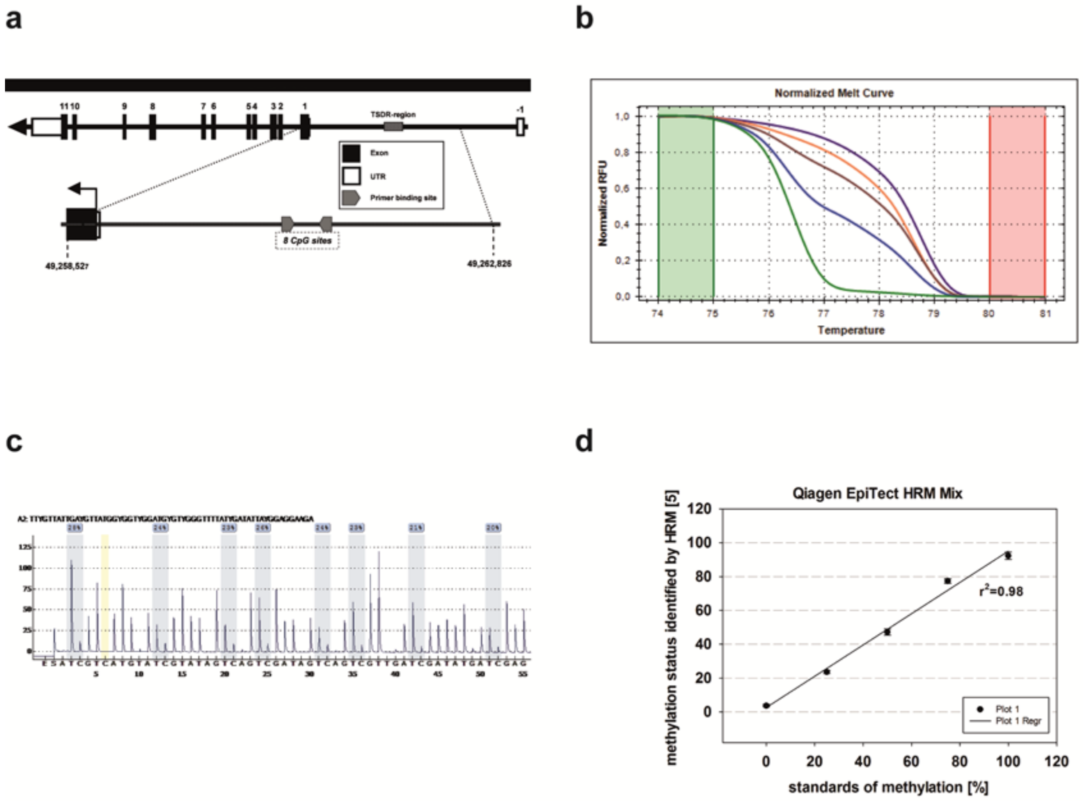

Supplementary Figure 14. DNA-methylation analysis of the human Foxp3 Treg-specific demethylated region (TSDR). (**a**) High resolution melting (HRM)-PCR primers were designed to cover the same area in the *Foxp3* locus (displayed transcript variant: ENST00000376207) as originally described by Baron *et al*. (2007). (**b**) Primers were validated on a standard-series of unmethylated and methylated DNA for unbiased PCR amplification according to their products' uniform high-resolution melting behavior. (**c**) The obtained amplicons enabled high-quality readouts of eight successive CpG-sites in subsequent pyrosequencing reactions (exemplary file shown). (**d**) High correlation between methylation levels of input standards and pyrosequencing-readout of PCR products additionally verified the designed assay for even amplification.

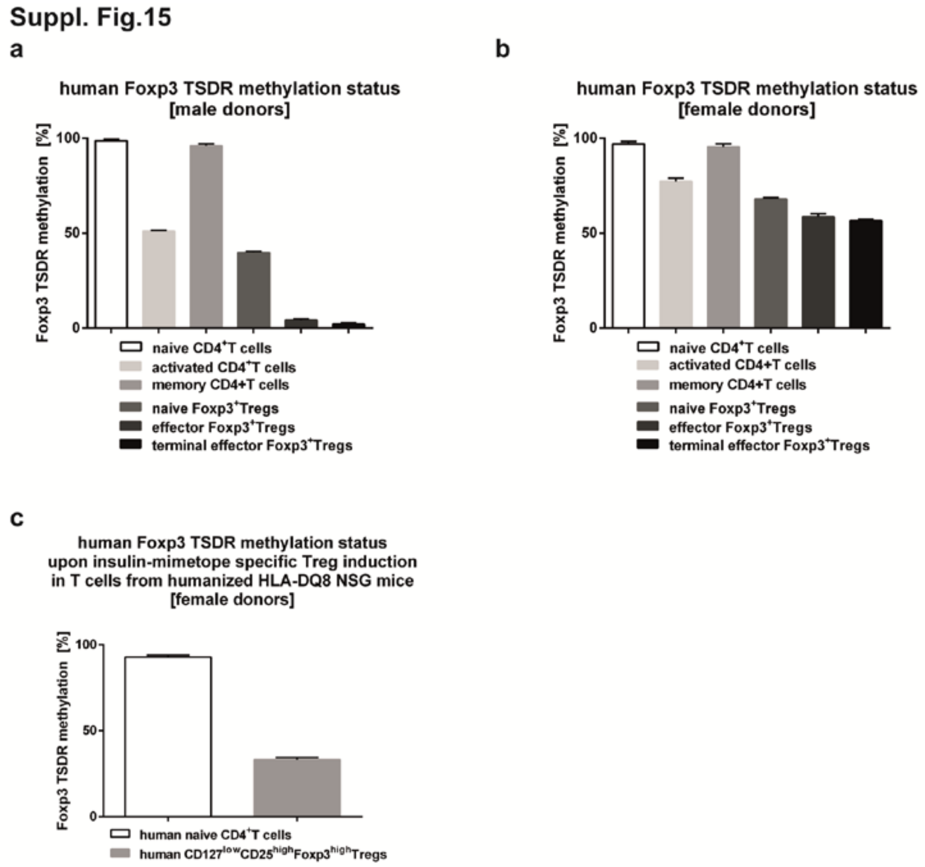

Supplementary Figure 15. DNA-methylation analysis of the human Foxp3 Treg-specific demethylated region (TSDR) in human CD4^{+}T cell subsets. Pyrosequencing analyses of eight successive CpG-sites in the human *Foxp3* TSDR region as methylation in %. Shown are means ± s.e.m. from female (**a**) or male (**b**) donors (n=4 each from two independent experiments). Except for the completely methylated cells the methylation levels of the female donor were higher than the levels of the male donor, which is due to the fact, that the *Foxp3* gene is X-linked. (**c**) Human *Foxp3* TSDR methylation status in naïve CD4^{+}T cells or CD4^{+}CD127^{low}CD25^{high}Tregs purified from pooled lymph nodes and spleens of humanized NSG-HLA-DQ8 transgenic mice (human female donors for reconstitution have been used) upon insulin mimetope-specific Treg induction (ins.mim.1= 14E-21G-22E and ins.mim.4=14E-21E-22E). Bars represent the means ± s.e.m from six mice and two independent experiments.

Suppl. Fig.16

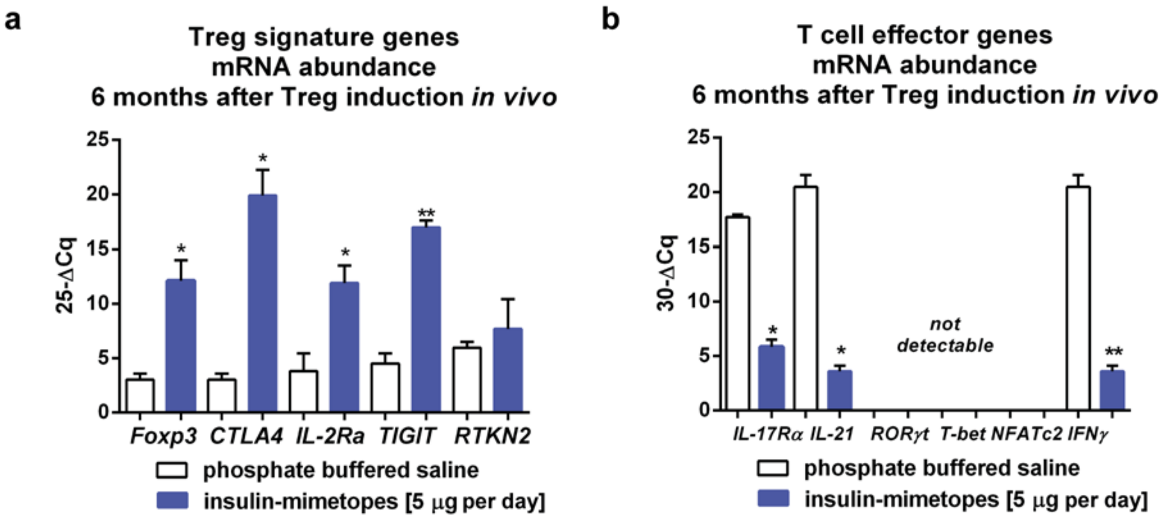

Supplementary Figure 16. Analysis of human Treg signature and T cell effector genes six months after subimmunogenic Treg induction with human insulin mimetopes in NSG-HLA-DQ8 transgenic mice. (a) Quantitative RT-PCR analyses of *Foxp3, CTLA4, IL-2R*α, *TIGIT* and *RTKN2* mRNA abundance in human CD4$^+$T cells purified from pooled spleens and lymph nodes of humanized mice six months after *in vivo* Treg induction using subcutaneous insulin mimetopes infusion by osmotic mini-pumps (ins.mim.1= 14E-21G-22E and ins.mim.4=14E-21E-22E) .Bars represent the means ± s.e.m. (n=5 mice per group). (**b**) Quantitative RT-PCR analyses of *IL-17R*α, *IL-21, ROR*γ*t, T-bet, IFN-*γ and *NFATc2* abundance in human CD4$^+$T cells purified from pooled spleens and lymph nodes of humanized mice six months after *in vivo* Treg induction using subcutaneous insulin mimetopes infusion by osmotic mini-pumps (ins.mim1= 14E-21G-22E and ins.mim4=14E-21E-22E) in humanized NSG-HLA-DQ8 transgenic mice (n=5). Bars represent the means ± s.e.m. (n=5 mice per group). * $P < 0.05$; ** $P < 0.01$ (Student´s *t*-test).

Suppl.Fig.17

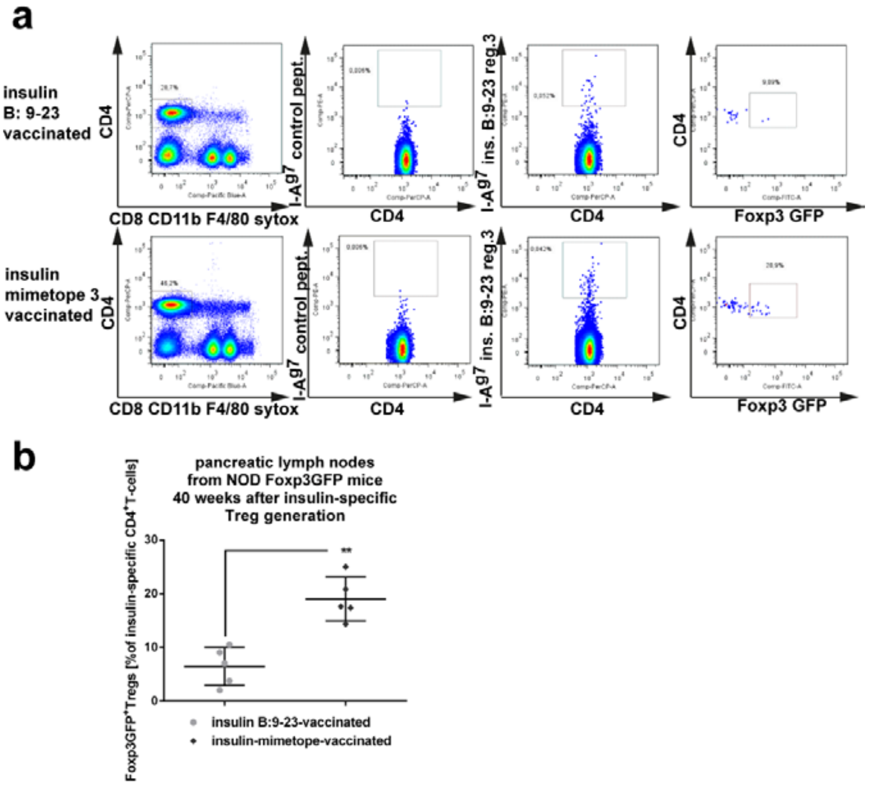

Supplementary Figure 17. Maintenance of insulin-specific Foxp3$^+$Tregs induced by subimmunogenic TCR stimulation and protection from type 1 diabetes development in Non-obese-diabetic (NOD) mice. Identification of I-A^{g7}-restricted insulin-specific CD4$^+$T cells by tetramer staining and flow cytometry, first gating on live, B220$^-$, F4/80$^-$, CD8$^-$, CD11b$^-$ and CD4, and then examining the tetramer binding (left panels). Control tetramer staining to assess specificity (second left panel). (**a**) Insulin-specific CD4$^+$T cells expressing Foxp3 GFP in pancreatic lymph nodes from 40-wk-old NOD-Foxp3 GFP reporter mice that had received the natural insulin B:9-23 epitope (upper panel) or the strong-agonistic insulin mimetope for Treg induction at 4-6 weeks of age (lower panel). (**b**) Percentages of Foxp3GFP$^+$Tregs in insulin-specific CD4$^+$T cells purified from pancreatic lymph nodes of 40-weeks-old NOD-Foxp3 GFP reporter mice. Data represent the means ± s.e.m., n=5 mice per group. ** $P < 0.01$ (Student´s *t*-test).

Suppl.Fig.18

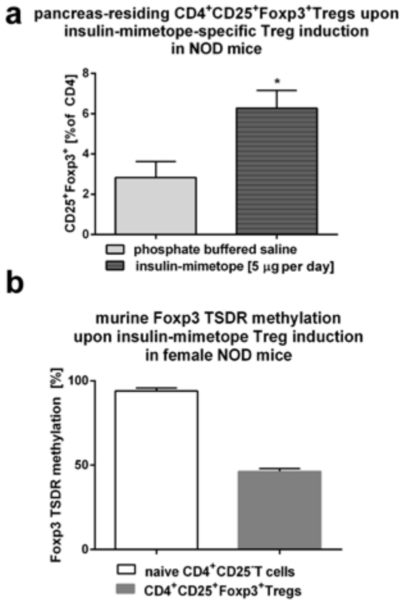

Supplementary Figure 18. Analyses of islet-residing CD4$^+$T cells purified from Non-obese-diabetic (NOD) mice upon insulin mimetope-specific Treg induction using subimmunogenic TCR stimulation. (a) Percentages of Foxp3$^+$CD4$^+$CD25$^+$Tregs in islet-infiltrating CD4$^+$T cells purified from NOD mice 4 weeks after Treg induction *in vivo*, n=6 mice per group. Data represent the means ± s.e.m., ** *P< 0.01* (Student´s *t*-test). (**b**) Pyrosequencing analyses of eight successive CpG-sites in the murine *Foxp3* TSDR region shown as methylation in %. Shown are means ± s.e.m. of islet-infiltrating naïve or Foxp3$^+$CD4$^+$CD5$^+$Tregs (n=6).
